# Internal fixation treatments for intertrochanteric fracture: a systematic review and meta-analysis of randomized evidence

**DOI:** 10.1038/srep18195

**Published:** 2015-12-11

**Authors:** Jiajie Yu, Chao Zhang, Ling Li, Joey S. W. Kwong, Li Xue, Xiantao Zeng, Li Tang, Youping Li, Xin Sun

**Affiliations:** 1 Chinese Evidence-based Medicine Center, West China Hospital, Sichuan University, Chengdu, China, 610041; 2 Center for Evidence-based Medicine and Clinical Research, Taihe Hospital, Hubei University of Medicine, Hubei, China, 442000; 3 Clinical Research and Evaluation Unit, West China Hospital, Sichuan University,610041; 4 Department of orthopedics, The third people’s hospital of Chengdu, Chengdu, China,610031; 5 Center for Evidence-based and Translational Medicine, Zhongnan Hospital, Wuhan University, Wuhan, China,430071; 6 School of Public Health, Curtin University, Perth, WA, Australia, 6845

## Abstract

The relative effects of internal fixation strategies for intertrochanteric fracture after operation remain uncertain. We conducted a systematic review and meta-analysis of randomized controlled trials (RCTs) to address this important issue. We searched PubMed, EMBASE and CENTRAL for RCTs that compared different internal fixation implants in patients with intertrochanteric fracture at 6-month follow-up or longer. We ultimately included 43 trials enrolling 6911 patients; most trials were small in sample sizes and events. Their risk of bias was generally unclear due to insufficient reporting. Because of these, no statistically significant differences were present from most of the comparisons across all the outcomes, and no definitive conclusions can be made. However, a number of trials compared two commonly used internal fixation strategies, gamma nail (GN) and sliding hip screw (SHS). There is good evidence suggesting that, compared to SHS, GN may increase the risk of cut out (OR = 1.87, 95% CI, 1.08 to 3.21), re-operation (OR = 1.61, 95% CI, 1.02 to 2.53), intra-operative (OR = 3.14, 95% CI, 1.34 to 7.35) and later fractures (OR = 3.67, 95% CI, 1.37 to 9.83). Future randomized trials or observational studies that are carefully designed and conducted are warranted to establish the effects of alternative internal fixation strategies for intertrochanteric fracture.

Hip fractures represent a common type of injuries; its number increases rapidly^1^. By 2050, the number of hip fractures is estimated to surpass 6.3 million^2^. The 1-year mortality for hip fractures range from 14% to 36%^3^. Hip fractures include femoral neck and intertrochanteric factures^4^; 20 to 30 percent of patients died in the first 12 months after an intertrochanteric fracture, especially those elderly with limited activity^5^,^6^. Surgical treatment represents the optimal strategy for managing intertrochanteric fractures. It allows early rehabilitation and functional recovery, and reduces the risk of postoperative complications^7^.

Internal fixation is a most common surgical treatment for intertrochanteric fractures^3^, and intramedually (nails) and extramedually (screws or plates) fixations are two commonly used approaches^8^. The established benefits of internal fixation treatments are immediate pain relief, rapid mobilization, accelerated rehabilitation and maintenance of independent living.

Several systematic reviews and meta-analysis have tested the effects of different internal fixation implants to provide insight into the options for treating intertrochanteric fractures from 2000 to 2012^9^,^10^,^11^,^12^,^13^. However, the findings in these studies are inconsistent and the diversity of devices used for intertrochanteric fractures had made it challenging for decision makers to identify the ideal treatment option. Meanwhile, the techniques and implants continue to be modified, which make the previous literature less relevant to current practice.

Therefore, we conducted a systematic review and meta-analysis, aiming to offer a comprehensive assessment of alternative internal fixation treatments for intertrochanteric fractures. The protocol of this study was registered on the PROSPERO database (CRD42014008795).

## Materials and Methods

### Study selection

We included prospective, randomized controlled trials (RCTs) published in English if they enrolled participants diagnosed with intertrochanteric fractures; compared currently used internal fixation implants; followed up patients for more than 6 months; and reported any of our pre-defined outcome measures of interest.

The currently available internal fixations included gamma nail (GN), ACE nail, holland nail, proximal femoral nail (PFN), proximal femoral nail antirotation (PFNA), intramedullary hip screw (IMHS), sliding hip screws (SHS), dynamic condylar screw (DCS), locking compression plate (LCP), percutaneous compression plate (PCCP), Medoff sliding plate, Targon proximal femoral and less invasive stabilization system (LISS).

The pre-defined outcomes included functional measures (i.e. quality of life scores, function scores), adverse events (i.e. mortality, cut out, non-union, reoperation, intra-operative fracture, later fracture, wound infection and embolism), and procedure measures (i.e. operative time, blood loss and hospital stay).

We excluded studies if patients had subtrochanteric fractures, pathological fractures, or previous femoral fractures.

### Data sources and searches

We searched PubMed, EMbase (via OVID) and the Cochrane Central Register of Controlled Trials (CENTRAL) from inception up to May, 2015 (Search strategy in appendix 1). We also searched ClinicalTrial.gov and the reference lists of included studies to identify additional eligible studies. To ensure completeness, we also cross referenced our search results with relevant published Cochrane systematic reviews of extracapsular hip fractures^14^.

### Study procedures

We used standardized pilot-tested data extraction forms for the screening of the abstracts and full texts, assessment of risk of bias and collection of data. Pairs of reviewers (YJJ, LL) independently screened study report for eligibility, assessed risk of bias and collected data from each eligible study. Discrepancies were resolved through discussion.

### Risk of bias assessment

We assessed the risk of bias of RCTs using a modified version of the Cochrane Collaboration’s tool^15^. The items included random sequence generation, allocation concealment, blinding of participants, surgeons or outcome assessors, completeness of outcome data, and selective reporting. We included two additional items regarding “standardization of the operative procedures” and “surgeons’ experience clearly defined with operations” because the validity of findings from surgical RCTs depends largely on quality of operation and surgeons’ experience^16^.

### Data extraction

We extracted the following data from each of the eligible studies: study characteristics (publish year, simple size, country, length of follow up), patient characteristics (gender, age, type of fracture); interventions (intramedullary and extramedullary treatments) and outcomes (quality of life scores, function scores, mortality, cut out, non-union, reoperation, operative fracture, later fracture, wound infection, embolism, operative time, blood loss and hospital stay)

### Data analyses

In the analysis of quality of life data, we reported the data at the baseline, end of the follow up, and the change from the baseline. For functional score data, because of the scanty in the reporting of the baseline data, we compared means at the end of follow up of those outcomes between treatment and control groups, assuming that the randomization has well achieved the balance of the baseline between groups. For each of the comparison, we pooled the quality of life data and the functional scores using weighted mean difference (MD) or standardized mean difference (SMD) if varying measures were used. In the analysis of the operation time, blood loss, and hospital stay, we treated each of the outcome measures as normally distributed, and pooled the mean differences for each of the comparison, and reported 95% confidence intervals. We also pooled, for each of the comparison, the trial data regarding adverse events.

In the meta-analyses, we applied the random-effects model using Mantel-Haenszel method. We examined heterogeneity by Cochran’s Q test and I^2^ statistic. Where possible, we conducted, for each meta-analysis, a pre-defined subgroup analysis by fracture types (stable fractures vs. unstable fractures by AO/OTA classification) to explore source of heterogeneity.

We performed sensitivity analyses by using alternative pooling methods (Peto method vs. Mantel-Haenszel method applicable to dichotomous data) and alterative statistical model (random vs. fixed effect). We performed the data analysis by the RevMan 5.3.

## Results

### Characteristics of included studies

**Figure 1:**
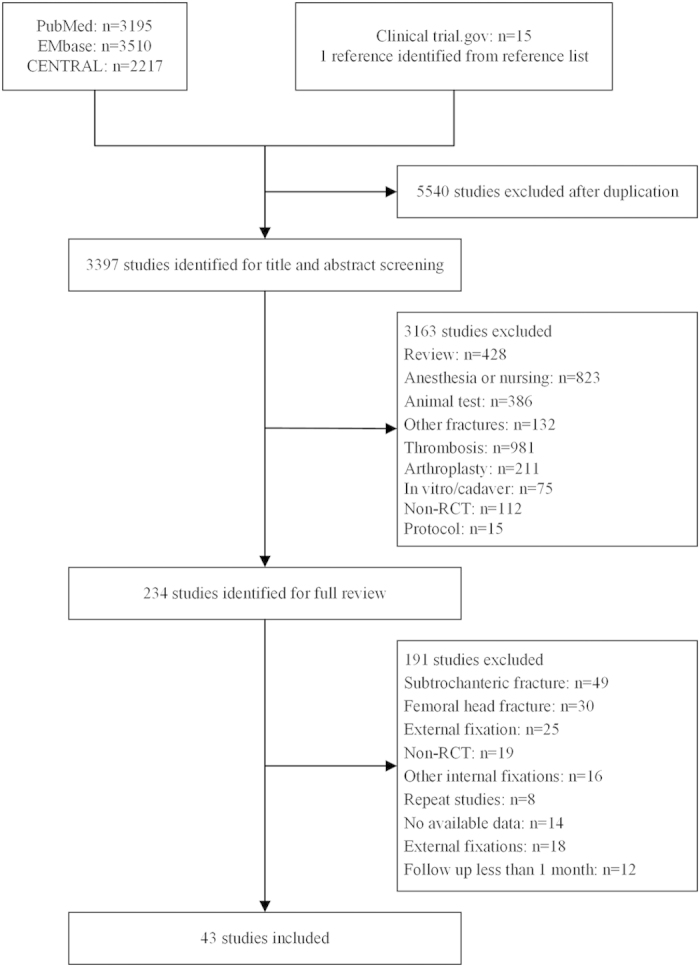
Flaw diagram of study selection based on the eligibility criteria. We initially searched 8937 reports and 3397 potential relevant reports were included in title and abstract screening after duplication (n = 5540). After screening of titles and abstracts, 234 records were retrieved for judging final eligibility. We eventually included 43 RCTs after full text reviewing.

The search yielded 3,397 potential relevant reports. After screening of titles and abstracts, 234 records were retrieved for judging final eligibility. We eventually included 43 RCTs involving 6911 patients (Fig. 1). These trials were conducted in 18 countries, of which 4 were international trials. The sample sizes ranged from 40 to 600, and the length of follow up from 6 to 40 months.

Among those trials, 52.4% (3625/6911) of the participants were female; the mean age ranged from 53.9 to 84.3 years; 30 trials (69.8%) recruited both stable and unstable fractures (n = 5010), 12 trials explored the effects of devices on unstable patients (n = 1695), and 1 trial did not report the fracture type of participants (n = 206) (Table 1).

Those 43 trials investigated 11 internal fixation treatments, including GN, ACE nail, holland nail, PFN, PFNA, IMHS, SHS, PCCP, Medoff sliding plate, Targon proximal femoral and LISS. The types of implants under assessment varied considerably across studies; few studies tested a same comparison. The details of comparisons were presented in Table 1.

Among those 43 trials, 14 (32.5%) adequately generated random sequences; 9 (20.9%) adequately concealed allocation; none blinded patients and surgeons; 10 (21.7%) blinded outcome assessors; 28 (65.1%) reported more than 80% of patients with completed follow up; only 1 (2.2%) did not report their pre-defined outcomes; 7 trials (15.2%) explicitly stated that surgeons standardized their operations by manuals or guidelines; and 31 (67.4%) referred that surgeons were experienced with trial operations (appendix 2).

### Effects on quality of life and functional measures

#### Quality of life scores (QoL scores)

Four trials (n = 420) used EuroQol 5D (EQ-5D) and Short Form (36) Health Survey (SF-36) to measure the effects of internal fixation treatments on the quality of life^17^,^18^,^19^,^20^, all of which were small in sample sizes. Two studies provided the baseline and follow up date on quality of life^17^,^19^; compared to the baseline, the scores at the end of follow up decreased. In the comparison of the data at the end of follow up, GN group had a significant higher score than SHS group in one trial reporting EQ-5D (MD: 0.12, 95%CI 0.02 to 0.22); in another small trial, no significant difference in the SF-36 was present between GN and PFNA (Table 2). The other two studies neither provided the data regarding standard deviation nor baseline.

#### Functional scores

Sixteen trials (n = 1467), consisting of 10 comparisons, reported functional status by 6 scores, including Parker-and-Palmer mobility score, Harris hip score, Jensen social-function score, Merle d’aubigne hip score, Geriatric hip fracture recovery scores, and Barthei index^17^,^19^,^21^,^22^,^23^,^24^,^25^,^26^,^27^,^28^,^29^,^30^,^31^,^32^. Ten studies provided the baseline and follow up date, all of which showed that functional scores decreased at the end of follow up^17^,^22^,^23^,^24^,^26^,^27^,^29^,^30^,^33^,^34^.

Eight trials compared GN versus 3 other devices (SHS, ACE nail and PFNA); 4 trials compared SHS versus other 4 devices (IMHS, PFN, PFNA, Medoff sliding plate); and 4 trials compared PFNA versus other 3 implants (LISS, Targon PF, PCCP) (appendix Fig. 1).

The comparison of means at the end of follow up showed that patients at GN group had a higher score compared to those at SHS group (SMD: 0.23, 95%CI 0.01 to 0.46, I^2^ = 22%), but had a lower score than PFNA group (SMD: −0.99, 95%CI −1.39 to −0.60, I^2^ = 66%). SHS had a higher score than IMHS (SMD: 0.43, 95%CI 0.03 to 0.83), but a lower score than PFNA (SMD: −0.73, 95%CI −1.18 to −0.29) (Table 3). No statistically significant differences were presented in other comparisons.

### Adverse events

#### Mortality

Thirty-three trials (n = 5940) reported mortality, all of which were at high risk of bias^17^,^18^,^20^,^21^,^22^,^24^,^27^,^28^,^29^,^30^,^31^,^32^,^33^,^34^,^35^,^36^,^37^,^38^,^39^,^40^,^41^,^42^,^43^,^44^,^45^,^46^,^47^,^48^,^49^,^50^,^51^,^52^,^53^. The mortality data were collected during the follow up of 6 to 40 months after operations. 14 trials compared GN versus 4 other implants (SHS, ACE nails, PFN, PCCP); 4 trials compared PFNA versus 3 other implants (LISS, Tragon PF, PCCP); and 17 trials compared SHS versus 7 other implants (appendix Figs 2–4). None of the comparisons showed statistically significant differences, likely due to the very small number of events and sample sizes across all the trials (Table 4).

#### Cut out

Twenty-nine trials (n = 3960)^13^,^14^,^15^,^16^,^17^,^18^,^19^,^21^,^22^,^23^,^24^,^25^,^26^,^28^,^34^,^35^,^36^,^37^,^41^,^42^,^44^,^46^,^47^,^48^,^49^,^52^,^54^,^55^,^56^ reported cut out data. Of those trials, 5 reported no event during the course of study (6–40 months)^17^,^26^,^30^,^38^,^57^. A total of 141 events were reported from 3692 patients (3.5%). Eighteen trials reported the comparison of GN versus 5 other implants (SHS, ACE nails, PFN, PFNA, PCCP); 11 trials compared SHS versus 6 other implants (appendix Figs 5–7); and 1 trial compared PFNA versus Targon PF.

The pooling of the trials showed that GN increased the risk of cut out compared to SHS (43/802 vs. 23/830; OR: 1.87, 95%CI 1.08 to 3.21, I^2^ = 0%). No statistically significant differences were found in other comparisons, largely because of the small number of events and sample sizes (Table 4).

#### Non-union

Data regarding non-union were available in 29 trials (n = 3795), among which 17 reported 90 non-union events during the follow up (2.37%)^17^,^19^,^24^,^26^,^27^,^28^,^29^,^30^,^34^,^35^,^36^,^37^,^38^,^39^,^40^,^41^,^42^,^44^,^47^,^48^,^49^,^50^,^51^,^52^,^53^,^54^,^57^,^58^,^59^. Fifteen trials compared GN with other 4 implants (SHS, ACE nails, PFN, PFNA); 12 tested the effect of SHS and other 7 devices; 2 compared PFNA with LISS and Targon PF (appendix Figs 8–10).

The pooling analysis suggested that SHS was associated with a lower risk of non-union compared to IMHS (1/228 vs. 9/246; OR: 0.15, 95%CI 0.03 to 0.87, I^2^ = 0%), but did not found statistically significant differences in other comparisons, again mostly because of the small number of events and sample sizes (Table 4).

#### Re-operation

Thirty-one trials (n = 4506) reported re-operation. A total of 248 re-operation events occurred in 4506 participants (5.5%)^17^,^18^,^21^,^22^,^26^,^27^,^29^,^30^,^32^,^33^,^34^,^35^,^36^,^37^,^38^,^39^,^40^,^41^,^42^,^44^,^45^,^46^,^48^,^49^,^50^,^51^,^53^,^54^,^56^,^57^,^59^. Sixteen trials compared GN with other 3 implants (SHS, PFN, PFNA); 14 compared SHS with other 7 implants; and 3 compared PFNA with PFN, LISS and Targon PF, respectively (appendix Figs 11–13).

Most of comparisons were made with few trials. The pooling of trials showed that GN increased the risk of re-operation compared to SHS (53/907 vs. 35/939; OR: 1.61, 95%CI 1.02 to 2.53, I^2^ = 0%) (Table 4). No statistically significant differences were found between the other comparisons.

#### Intra-operative fracture

A total of 17 trials (n = 2661) reported intra-operative fracture data^17^,^21^,^22^,^27^,^29^,^30^,^34^,^36^,^37^,^38^,^39^,^40^,^41^,^42^,^47^,^49^,^54^. Of those, 59 intra-operative fractures occurred during the follow up (2.21%). Five comparisons were made among those trials, including the comparisons between GN versus SHS, GN versus PFN, SHS versus IMHS, SHS versus PFN, and SHS versus PFNA (appendix Figs 14,15).

Most of the comparisons did not demonstrated statistically significant differences because of the small number of events. However, the pooling of the trials showed increased risk of intra-operative fracture in patients receiving GN versus those with SHS (22/861 vs. 5/861; OR: 3.14, 95%CI 1.34 to 7.35, I^2^ = 0%), and in patients with GN versus with PFN (19/125 vs. 5/125; OR: 4.30, 95%CI 1.55 to 11.92) (Table 4).

#### Later fracture

Twenty-six trials, totaling 3508 patients and 42 events, reported data on later fracture rate^17^,^19^,^21^,^22^,^26^,^29^,^30^,^33^,^34^,^36^,^37^,^38^,^39^,^40^,^41^,^42^,^44^,^47^,^50^,^51^,^53^,^54^,^56^,^57^,^58^,^59^. Sixteen trials compared GN with other 3 implants; 9 compared SHS with other 6 devices; 1 compared PFNA with PFN; and the other 1 compared PFNA with Targon PF (appendix Figs 16–18).

Again, most of the comparisons did not show statistically significant differences between the alternative surgical options. Pooling of the trials comparing GN versus SHS, however, showed that GN was associated with increased risk of later fractures (18/703 vs. 2/704; OR 3.67, 95%CI 1.37 to 9.83, I^2^ = 0%) (Table 4).

#### Wound infection

Thirty trials reported wound infections (superficial infections and deep infections) (n = 4265)^18^,^19^,^21^,^22^,^23^,^24^,^25^,^26^,^27^,^29^,^30^,^32^,^33^,^34^,^36^,^38^,^39^,^40^,^41^,^42^,^45^,^46^,^48^,^50^,^51^,^53^,^54^,^57^,^58^,^59^. Among those, a total of 147 patients (3.4%) reported to have wound infection events. Eighteen trials compared GN with other 4 implants (SHS, ACE nails, PFN, PFNA); 11 compared SHS with 7 devices; and 2 compared PFNA with LISS and Targon PF (appendix Figs 19–21). None of the comparisons showed statistically significant differences in the risk of wound infection (Table 4).

#### Embolism

A total of 21 trials (n = 2655) provided data on embolism (deep venous embolism and pulmonary embolism), and 88 embolic events occurred in 2657 participants (3.3%)^21^,^22^,^23^,^24^,^27^,^29^,^31^,^33^,^36^,^37^,^41^,^42^,^45^,^46^,^48^,^50^,^54^,^59^. Eleven trials reported the comparative outcomes of GN and 3 other implants, 7 trials tested the effect of SHS and other 5 devices; and one trial compared PFNA with Targon PF (appendix Figs 22–24). Of 9 comparative groups, SHS increased the risk of embolism compared to PCCP (11/87 vs. 4/92; OR: 3.40, 95%CI 1.02 to 11.26, I^2^ = 0%) (Table 4).

### Procedure measures

#### Operative time (min)

Differences between internal fixation treatments on operation duration were reported in 34 trials including 5692 patients^[17–19,22–26,29,30,32,34–36,38–42,46–53,55–59^. Heterogeneity across the studies were high in some comparisons. 15 trials compared GN with other 4 implants (SHS, ACE nails, PFN, PFNA); 16 trials compared SHS with 7 other devices; and 3 trials compared PFNA with 3 implants (appendix Figs 25–27). We found a substantial difference on operative time among varied comparisons, likely due to different definitions in original trials. Overall, PFNA were associated with less operative time than other internal fixation treatments (PFNA vs. GN: (MD:−4.45, 95%CI −5.17 to −3.73, I^2^ = 0%); PFNA vs. LISS: (MD: −26.78, 95%CI −32.8 to −20.75, I^2^ = 0%); PFNA vs. Targon PF: (MD: −18.5, 95%CI −30.63 to −6.37); PFNA vs. PCCP: (MD:13.5, 95%CI 7.54 to19.46)) (Table 5).

#### Blood loss (mL)

Data on blood loss were available in 19 trials (n = 3475)^20^,^21^,^25^,^26^,^27^,^30^,^31^,^32^,^33^,^35^,^39^,^46^,^47^,^48^,^50^,^52^,^54^,^55^,^57^. Heterogeneity across the studies were high in some comparisons. 7 trials compared GN with other 4 implants (SHS, ACE nails, PFN, PFNA); 9 trials compared SHS with 5 other devices; and 3 trials compared PFNA with LISS and PCCP (appendix Figs 28–30). Overall, patients who underwent SHS had more blood loss than those who were treated with other internal fixation implants (SHS vs. IMHS: (MD: 62.42, 95%CI 26.28 to 98.56, I^2^ = 0%); SHS vs. PFNA: (MD: 253.86, 95%CI 237.47 to 270.25, I^2^ = 0%); SHS vs. Holland nail: (MD: 82.0, 95%CI 37.81 to 126.91) (Table 5).

#### Hospital stay (days)

A total of 22 trials (n = 3705) reported the duration of hospital stay^18^,^19^,^26^,^29^,^30^,^31^,^32^,^33^,^34^,^35^,^37^,^38^,^39^,^40^,^43^,^45^,^46^,^47^,^48^,^51^,^52^,^54^. 11 trials compared GN with other 4 implants (SHS, ACE nails, PFN, PCCP); 9 trials compared SHS with 6 other devices; and 3 trials compared PFNA with LISS and PCCP (appendix Figs 31–33). One comparison revealed that PFN had longer hospital stay compared to GN (MD: 2.7, 95%CI 2.44 to 2.96), and no significant differences were found in other 11 comparisons of devices (Table 5).

### Sensitivity analysis

The sensitivity analyses using alternative analysis methods (Peto method vs. Mantel-Haenszel method), and considerations of heterogeneity (random-effects vs. fixed-effect) did not show important changes in the pooled effects for these outcomes.

## Discussion

Our study has identified a wide variety of internal fixation implants for patients with intertrochanteric fracture, among which sliding hip screw (SHS) and gamma nail (GN) were the most commonly investigated treatment options, as evident from the trials. The other implants, including percutaneous compression plate (PCCP), proximal femoral nail antirotation (PFNA), and proximal femoral nail (PFN), also are often investigated.

The findings from those trials suggested that substantial uncertainty regarding the relative effects – both benefits and harms – remain among those alternative internal fixation strategies, except only a few comparisons, because of the small number of sample sizes with the vast majority of trials and the serious limitations that threat the validity (e.g. failure to conceal treatment allocation).

The quality of life and functional status are of important interest to surgical treatment and are often used in orthopedics surgical trials^60^,^61^,^62^,^63^,^64^. However, our review identified under-reporting of these outcomes. In the trials having reported these two outcomes, the completeness of data remains less satisfied in most circumstances – many trials failed to report the baseline data and the change from baseline; even if reported, the standard deviations were not available. All those limitations have made fair comparison of alternative internal fixation strategies less likely. Given the current body of evidence, it is uncertain if the quality of life would be improved after the surgical interventions, and which of surgical treatment would achieve better quality of life. In our analyses of the functional scores, the findings similarly suggested a lack of evidence, and no definitive conclusions can be made for most of the comparisons. However, it seems from the analyses that patients receiving PFNA might achieve better functional status after surgery than those receiving GN or SHS. This finding was preliminary given the limitations of the included trials.

The trials extensively reported complications of internal fixation treatments. However, due to the small sample sizes and methodological limitations, the current body of evidence is inadequate to draw clear conclusion for most of the comparisons. The analyses suggested substantial uncertainty of relative effects on complications between internal fixation strategies. However, a number of trials compared GN and SHS. The analyses consistently suggested that patients receiving GN may have significantly higher risk of complications than those SHS, including the risk of cut out, operations, intra-operative fracture, and later facture. The consistency of findings across studies and outcomes, and the relatively large magnitude of effect increases the credibility of this finding. A few other studies also suggested that SHS might have a lower risk of non-union, but have a higher risk of embolism than PCCP. These findings were, however, fragile given the small number of events and sample sizes, as well as the potential risk of bias those studies pose.

A number of trials also reported procedure measures (operation time, blood loss, and hospital stay). The findings were however inconsistent across studies, which resulted in substantial heterogeneity. The presence of the varying procedural outcomes across studies may represent the differential levels of expertise among the surgeons participating in those trials. Overall, the results suggested patients undergoing PFNA may have shorter operative time, and patients undergoing SHS may have more blood loss than other extramural implants.

Compared to the Cochrane systematic review that compared intramedullary nails with SHS for extracapsular hip fractures^14^, we excluded subtrochanteric fractures and assessed more extramedullary implants. The Cochrane Review conducted in 2010 included 43 RCTs that set no limit regarding the length of follow up. They found that the SHS was a better fixation device for the intertrochanteric fractures than nails. They also suggested intramedullary nails have advantages over extramedullary plates/screws for some unstable intertrochanteric fractures. Considering a wide variety of implants with inconsistent outcomes and low precision of estimate effects, we suggest that cautions need to be taken in drawing any definite conclusions.

We conducted a comprehensive systematic review using rigorous methods. However, there are a few limitations. First, because of the limited availability of data, we compared means of functional scores at the end of follow up between treatment groups. We assumed that the data at baseline were well balanced between groups. However, this assumption may not always be hold. Second, the trials we included in the analysis suffered from important methodological limitations, as many other surgical trials. The potential risk of bias that those trials poses has weakened our inference of the treatment effects. Third, most of the trials included in our analyses were small in sample sizes. This has resulted in imprecise estimation of effects, and definitive conclusion is unlikely for most of the comparisons. Fourth, due to the limited evidence with different types of fractures (69.7% of studies did not take into account of the fracture stability), we were unable to explore if the treatment effects might differ by fracture types.

In conclusion, due to the small number of events and sample sizes and serious limitations that those trials pose, the current body of evidence is inadequate to establish the relative effects – including quality of life, functional scores, and complications – of all of the alternative internal fixation strategies. However, the evidence suggests that patients undergoing GN may have a higher risk of complications than those receiving SHS. Future trials that are adequately powered and well designed and conducted are warranted to fairly test the effects of the different surgical treatments. Observational studies that are carefully collect and analyze the data may also provide important insights regarding the effects of the surgical treatments.

## Additional Information

**How to cite this article**: Yu, J. *et al.* Internal fixation treatments for intertrochanteric fracture: a systematic review and meta-analysis of randomized evidence. *Sci. Rep.* **5**, 18195; doi: 10.1038/srep18195 (2015).

## Supplementary Material

Supplementary Information

## Figures and Tables

**Table 1:** Characteristics of included studies

Study	Country	International (Y/N)	Intervention		n		Male/Female		Age (Mean (SD))		Stable fracture		Unstable fracture		Follow up (month)
			E	C	E	C	E	C	E	C	E	C	E	C	
**Gamma nails vs. others**															
Adams, 2001	UK	N	GN	SHS	203	197	39/164	49/148	81.2 (8.5)	80.7 (11.7)	111	96	92	101	12
Ahrengart, 2001	Sweden	Y	GN	SHS	210	216	62/148	61/155	80.5 (NR)	79.0 (NR)	107	144	103	72	6
Aktselis, 2014	Greece	N	GN	SHS	40	40	12/28	12/28	82.9 (5.8)	83.1 (6.5)	0	0	40	40	12
Barton, 2010	UK	N	GN	SHS	100	110	19/81	25/85	83.1 (9.5)	83.3 (6.8)	0	0	100	110	12
Bridle, 1991	UK	N	GN	SHS	49	51	9/40	7/44	82.7 (NR)	81 (NR)	18	23	31	28	6
Hoffman, 1996	Newzealand	N	GN	SHS	31	36	4/27	12/24	83.2 (8.1)	79.0 (10.4)	21	24	10	12	6
Kukla, 1997	Austria	N	GN	SHS	60	60	14/46	4/56	83.0 (9.1)	84.0 (8.3)	31	23	29	37	6
Leung, 1992	HongKong	N	GN	SHS	113	113	25/68	30/63	80.8 (8.4)	78.3 (9.5)	30	20	63	73	12
O’Brien, 1995	Canada	N	GN	SHS	52	49	9/43	17/32	83.0 (9.5)	77.0 (13.7)	30	28	23	21	12
Ovesen, 2006	Darmark	N	GN	SHS	73	73	20/53	21/52	79.9 (10)	78.5 (11.7)	23	17	50	56	12
Park, 1998	Korea	N	GN	SHS	30	30	10/20	14/16	73.7 (NR)	72.2 (NR)	14	11	16	19	18
Radford, 1993	UK	N	GN	SHS	100	100	79/21	76/24	83 (6.2)	78.0 (5.0)	38	57	62	43	12
Utrilla, 2004	Spain	N	GN	SHS	104	106	38/66	28/78	80.6 (7.5)	79.8 (7.3)	81	75	23	31	12
Efstathopoulos, 2007	Greece	N	GN	ACE	56	56	19/37	13/43	79.5 (NR)	78.1 (NR)	12	8	44	48	8
Grave, 2012	Belgium	N	GN	ACE	61	51	35/26	32/19	73.0 (12.5)	77.0 (14.0)	18	20	43	31	12
Vidyadhara, 2007	India	N	GN	ACE	37	36	19/18	18/18	68.6 (5.6)	69.4 (4.6)	0	0	37	36	24
Herrera, 2002	Spain	N	GN	PFN	125	125	71/179		78.9(NR)		13	19	112	98	12
Schipper, 2004	Netherland	Y	GN	PFN	213	211	37/176	38/173	82.6 (NR)	82.2 (NR)	165	156	48	55	12
Xu, 2010 (1)	China	N	GN	PFNA	70	66	27/43	27/39	75.4 (1.0)	76.0 (1.2)	0	0	70	66	17
Vaquero, 2012	Spain	N	GN	PFNA	31	33	5/25	3/28	83.5 (7.4)	83.6 (7.5)	0	0	31	33	12
VarelaEgocheaga, 2009	Spain	N	GN	PCCP	40	40	6/34	11/29	82.5 (NR)	81.6 (NR)	27	24	13	16	12
**SHS vs. others**															
Janzing, 2001	Netherland	N	SHS	PCCP	44	39	10/34	4/35	83.0 (8.5)	82.0 (7.7)	25	16	19	23	12
Kosygan, 2002	UK	N	SHS	PCCP	56	55	12/44	9/46	82.8 (9.0)	82.7 (8.5)	25	25	31	30	6
Peyser, 2007	Israel	N	SHS	PCCP	53	50	18/35	16/34	82.5 (8.0)	78.9 (8.2)	31	29	14	8	12
Yang, 2011	USA	N	SHS	PCCP	33	33	7/25	11/22	77 (14.2)	76 (17.5)	0	0	33	33	12
Baumgaertner, 1998	USA	N	SHS	IMHS	68	67	45/86		79 (9.8)		35	31	33	36	28
Hardy, 1998	Belgium	N	SHS	IMHS	50	50	15/35	8/42	79.5 (10.7)	81.7 (11.8)	16	13	34	37	12
Harrington, 2002	USA	N	SHS	IMHS	52	50	11/41	9/41	82.1 (8.6)	83.8 (8.5)	0	0	52	50	12
McCormack, 2013	Canada	Y	SHS	Medoff	86	77	21/65	18/59	83.0 (NR)	83.6 (NR)	0	0	86	77	6
Watson, 1998	USA	N	SHS	Medoff	91	69	43/117	76 (12.3)	29	17	62	52	16		
Lunsjo, 2001	Sweden	Y	SHS	Medoff	238	268	71/167	67/201	81 (7.8)	81 (9.5)	0	0	238	268	12
Saudan, 2002	Switzerland	N	SHS	PFN	106	100	22/84	24/76	83.7 (10.1)	83.0 (9.7)	NR				12
Garg,2011	India	N	SHS	PFNA	39	42	27/12	32/10	64.3 (4.5)	60.2 (5.0)	0	0	39	42	40
Xu, 2010(2)	China	N	SHS	PFNA	55	51	16/39	15/36	77.9 (7.8)	78.5 (8.0)	0	0	55	51	12
Zou, 2009	China	N	SHS	PFNA	63	58	15/48	12/46	65 (13.7)	65 (13.5)	52	42	11	16	12
Little, 2008	UK	N	SHS	Holland	92	98	8/84	20/78	82.6 (8.0)	84.2 (8.0)	15	29	77	69	12
Parker, 2012	UK	N	SHS	Targon PF	300	300	52/248	69/231	82.4 (13)	81.4 (12.8)	58	65	242	235	12
**PFNA vs. others**															
Guo, 2013	China	N	PFNA	PCCP	45	45	16/29	19/26	74.2 (8.8)	71.6 (7.5)	22	18	23	27	12
Park, 2010	Korea	N	PFNA	PFN	23	17	6/17	3/14	75.7 (6.7)	67.0 (11.0)	7	5	16	12	24
Tao, 2013	China	N	PFNA	LISS	45	42	16/29	17/25	80.4 (7.3)	79.6 (7.6)	10	9	35	33	12
Zhou, 2011	China	N	PFNA	LISS	36	28	17/19	13/15	76.2 (15.2)	67.8 (15.7)	8	3	28	25	26
Wild, 2010	Germany	N	PFNA	Targon PF	40	40	20/20	20/20	81.8 (8.5)	83.1 (11.7)	29	17	62	52	12
**Others**															
Papasimos, 2005	Greece	N	GN	SHS	40	40	16/24	14/26	82.8 (NR)	81.4 (NR)	0	0	40	40	12
			PFN		40		17/23		79.4 (NR)		0	0	40		

Y: Yes; N: No; n: number of patients; GN: Gamma Nail; ACE: ACE nails; IMHS: Intramedullary Hip Screw; SHS: Sliding Hip Screw; PFN: Proximal Femoral Nail; PFNA: Proximal Femoral Nail Antirotation; Medoff: Medoff sliding plate; PCCP: Percutaneous Compression Plate; Targon PF: Targon Proximal Femoral; LISS: Less Invasive Stabilization System.

**Table 2:** Results of quality of life.

Study	Intervention	Tool	No of pts at BL and FU	Baseline Mean (SD)	Follow up bMean (SD)	MD (95%CI)
Aktselis, 2014	GN	EuroQol 5D	40/36	0.92 (0.14)	0.90 (0.16)	0.12 (0.02,0.22)
	SHS		40/35	0.90 (0.14)	0.78 (0.27)	
Barton, 2010	GN	EuroQol 5D	100/65	NR	0.46 (NR)	—
	SHS		110/86	NR	0.37 (NR)	
Vaquero, 2012	GN	SF-36	28/11	PCS:39.7 (7.9)	35.0 (10.8)	PCS:
				MCS:51.7 (9.8)	46.3 (12.8)	−1.60 (−11.54,8.34)
	PFNA		27/10	PCS:42.0 (8.1)	36.6 (12.3)	MCS:
				MCS:46.9 (11.2)	47.8 (11.3)	−1.50 (−11.81,8.81)
Yang, 2011	PCCP	SF-36	33/20	NR	47.50 (NR)	—
	SHS		33/18	NR	38.60 (NR)	

GN: Gamma Nail; IMHS: Intramedullary Hip Screw; SHS: Sliding Hip Screw; PCCP: Percutaneous Compression Plate; AMBI: AMBI hip screw; PCS: Physical component summary score; MCS: Mental component summary score

Pts: patients; BL: baseline; FU: follow up; NR: Not reported;

—: Not applicable.

**Table 3:** Meta-analysis of functional scores.

Intervention	N (n)	SMD (95%CI)	I^2^ (%)
**GN vs.**			
SHS	3 (305)	0.23 (0.01,0.46)*	22
ACE nail	3 (273)	−0.16 (−0.40,0.08)	61
PFNA	2 (114)	−0.99 (−1.39,−0.60)*	66
**SHS vs.**			
IMHS	1 (100)	0.43 (0.03,0.82)*	—
PFN	1 (168)	0.06(−0.24.0.37)	—
PFNA	1 (83)	−0.73 (−1.18,−0.29)*	—
Medoff	1 (125)	−0.07 (−0.42,0.28)	—
**PFNA vs.**			
LISS	2 (151)	−0.01 (−0.33,0.31)	0
Targon PF	1 (58)	0.06 (−0.45,0.58)	0
PCCP	1 (90)	−0.09 (−0.50,0.32)	—

SMD: standard mean difference; 95% CI: 95% confidence interval.

GN: Gamma Nail; IMHS: Intramedullary Hip Screw; SHS: Sliding Hip Screw; PFN: Proximal Femoral Nail; PFNA: Proximal Femoral Nail Antirotation; PCCP: Percutaneous Compression Plate; Targon PF: Targon Proximal Femoral; LISS: Less Invasive Stabilization System.

*Statistically significant differences.

—:Not applicable.

**Table 4:** Meta-analysis of adverse events.

Intervention	Mortality				Cut out				Non-union			
	N (n)	Events/total	OR (95%CI)	I^2^	N (n)	Events/total	OR (95%CI)	I^2^	N (n)	Events/total	OR (95%CI)	I^2^
**GN vs.**												
SHS	11 (2185)	229/1083 vs. 219/1102	1.08(0.88,1.34)	0%	12 (1632)	43/802 vs. 23/830	1.87 (1.08,3.21)*	0%	11 (1348)	32/566 vs. 27/574	1.21 (0.72,2.06)	0%
ACE nails	1 (112)	14/61 vs. 12/51	0.97(0.40,2.33)	—	1 (73)	1/37 vs. 0/36	3.00 (0.12,76.09)	—	1 (85)	0/47 vs. 0/38	—	—
PFN	2 (674)	66/338 vs. 75/336	0.84(0.58,1.22)	0%	2 (508)	18/261 vs. 12/247	1.64 (0.48,5.55)	32%	1 (197)	1/101 vs. 2/96	0.47 (0.04,5.27)	—
PFNA	—	—	—	—	2 (154)	0/77 vs. 3/77	0.13 (0.01,2.70)	—	2 (154)	2/77 vs. 3/77	0.67 (0.10,4.30)	—
PCCP	1 (80)	1/40 vs. 4/40	0.23(0.02,2.16)	—	1 (75)	2/39 vs. 0/36	4.87 (0.23,104.88)	—	—	—	—	—
**SHS vs.**												
PCCP	5 (466)	46/242 vs. 29/224	1.53(0.91,2.58)	1%	2 (179)	4/87 vs. 2/92	2.18 (0.39,12.19)	0%	1 (83)	0/44 vs. 0/39	—	—
IMHS	3 (337)	52/170 vs. 46/167	1.15(0.71,1.87)	0%	2 (208)	4/106 vs. 3/102	1.30 (0.28,6.04)	0%	3 (238)	2/121 vs. 2/117	0.96 (0.16,5.67)	—
Medoff	3 (783)	87/384 vs. 77/399	1.25(0.89,1.78)	0%	1 (160)	4/91 vs. 1/69	3.13 (0.34,28.62)	—	2 (474)	1/228 vs. 9/246	0.15 (0.03,0.87)*	0%
PFN	2 (314)	15/160 vs. 20/154	0.68(0.33,1.40)	0%	2 (248)	3/119 vs. 4/129	0.82 (0.15,4.42)	2%	1 (168)	0/89 vs. 0/79	—	—
PFNA	2 (187)	8/94 vs. 8/93	1.0(0.35,2,87)	0%	3 (277)	6/142 vs. 0/135	15.98 (0.87,295.14)	—	3 (277)	1/142 vs. 0/135	2.81 (0.11,70.31)	—
Targon PF	1 (600)	81/300 vs. 83/300	0.97(0.68,1.38)	—	1 (430)	3/215 vs. 2/215	1.51 (0.25,9.11)	—	1 (230)	1/215 vs. 1/215	1.00 (0.06,16.09)	—
Holland nail	1 (190)	18/98 vs. 16/92	1.07(0.51,2.25)	—	—	—	—	—	1 (156)	0/80 vs. 0/76	—	—
**PFNA vs.**												
LISS	2 (164)	6/85 vs. 5/78	1.10(0.32,3.77)	0%	—	—	—	—	1 (87)	0/45 vs. 0/42	—	—
Targon PF	1 (80)	9/40 vs. 9/40	1.0(0.35,2.86)	—	1 (58)	3/29 vs. 2/29	1.56 (0.24, 10.09)	—	1 (58)	0/29 vs. 1/29	0.32 (0.01,8.24)	—
PCCP	1 (61)	0/30 vs. 0/31	Not estimable	—	—	—	—	—	—	—	—	—
**Intervention**	**Re-operation**				**Intra-operative fracture**				**Later fracture**			
	**N (n)**	**events/total**	**OR(95%CI)**	**I^2^**	**N (n)**	**events/total**	**OR (95%CI)**	**I^2^**	**N (n)**	**events/total**	**OR (95%CI)**	**I^2^**
**GN vs.**												
SHS	13 (1846)	53/907 vs. 35/939	1.61(1.02,2.53)*	0%	11 (1722)	22/861 vs. 5/861	3.14 (1.34,7.35)*	0%	12 (1407)	18/703 vs. 2/704	3.67 (1.37,9.83)*	0%
PFN	2 (511)	30/261 vs. 35/250	0.86(0.40,1.84)	40%	1 (250)	19/125 vs. 5/125	4.30 (1.55,11.92)*		2 (511)	5/261 vs. 4/250	1.27 (0.03,46.52)	74%
PFNA	1 (93)	0/47 vs. 1/46	0.32(0.01,8.04)	—	—	—	—	—	2 (154)	2/77 vs. 3/77	0.75 (0.08,6.83)	24%
**SHS vs.**												
PCCP	3 (261)	13/131 vs. 7/130	1.69(0.32,8.96)	61%	—	—	—	—	1 (83)	0/44 vs. 0/39	—	—
IMHS	1 (70)	4/35 vs. 3/35	1.38(0.28,6.66)	—	3 (337)	0/170 vs. 6/167	0.20 (0.03,1.17)	0%	2 (168)	0/86 vs. 0/82	—	—
Medoff	2 (474)	8/228 vs. 11/246	1.25(0.24,6.39)	63%	—	—	—	—	—	—	—	—
PFN	2 (248)	5/129 vs. 11/119	0.76(0.10,5.62)	64%	2 (286)	0/146 vs. 0/140	—	—	2 (247)	0/128vs. 1/119	0.33 (0.01,8.22)	—
PFNA	3 (300)	10/154 vs. 2/146	3.21(0.34,30.55)	50%	1 (106)	0/55 vs. 2/51	0.18 (0.01,3.81)	—	2 (204)	0/106 vs. 1/98	0.30 (0.01,7.65)	—
Targon PF	1 (430)	9/215 vs. 3/215	3.09(0.82,11.56)	—	—	—	—	—	1 (430)	0/215 vs. 1/215	0.33 (0.01,8.19)	—
Holland nail	1 (156)	1/80 vs. 0/76	2.89(0.12,71.96)	—	—	—	—	—	1 (156)	0/80 vs. 0/76	—	—
**PFNA vs.**												
PFN	1 (40)	0/17 vs. 1/23	0.43(0.02,11.18)	—	—	—	—	—	1 (40)	0/17 vs. 1/23	0.43 (0.02,11.18)	—
LISS	1 (59)	1/34 vs. 2/25	0.35(0.03,4.07)	—	—	—	—	—	—	—	—	—
Targon PF	1 (58)	4/29 vs. 2/29	2.16(0.36,12.84)	—	—	—	—	—	1 (58)	1/29 vs. 0/29	3.11 (0.12,79.43)	—
**Intervention**	**Wound infection**				**Embolism**							
	**N (n)**	**events/total**	**OR(95%CI)**	**I^2^**	**N (n)**	**events/total**	**OR (95%CI)**	**I^2^**				
**GN vs.**												
SHS	11 (1433)	20/710 vs. 22/723	0.94(0.49,1.79)	0%	7 (886)	27/440 vs. 24/446	1.08 (0.61,1.92)	0%				
ACE nails	3 (246)	3/125 vs. 4/121	0.85(0.18,4.04)	—	2 (173)	1/88 vs. 0/85	3.52 (0.14,88.76)	—				
PFN	2 (511)	25/261 vs. 17/250	1.46(0.76,2.79)	0%	2 (511)	2/261 vs. 6/250	0.32 (0.06,1.64)	0%				
PFNA	2 (197)	1/100 vs. 5/97	0.28(0.04,1.87)	0%	—	—	—	—				
**SHS vs.**												
PCCP	2 ((179)	2/87 vs. 0/92	3.25(0.33,31.88)	0%	2 (179)	11/87 vs. 4/92	3.40 (1.02,11.26)*	0%				
IMHS	1 ((70)	0/35 vs. 0/35	—	—	1 (70)	3/35 vs. 1/35	3.19 (0.32,32.24)	—				
Medoff	2 (474)	5/228 vs. 2/246	2.41(0.53,10.90)	0%	1 (349)	1/165 vs. 0/184	3.36 (0.14,83.17)	—				
PFN	2 (248)	2/129 vs. 4/119	0.47(0.08,2.78)	—	2 (248)	6/129 vs. 4/119	1.44 (0.39,5.37)	0%				
PFNA	2 (204)	4/106 vs. 2/98	1.83(0.31,10.84)	0%	—	—	—	—				
Targon PF	1 (430)	4/215 vs. 4/215	1.00(0.25,4.05)	—	—	—	—	—				
Holland nail	1 (156)	10/80 vs. 5/76	2.03(0.66,6.24)	—	1 (156)	1/80 vs. 0/76	2.89 (0.12,71.96)	—				
**PFNA vs.**												
LISS	1 (59)	0/34 vs. 0/25	—	—	1 (87)	1/45 vs. 0/42	2.87 (0.11,72.29)					
Targon PF	1 (58)	4/29 vs. 2/29	2.16(0.36,12.84)	—	—	—	—	—				

N: the number of studies; n: number of patients.

GN: Gamma Nail; IMHS: Intramedullary Hip Screw; SHS: Sliding Hip Screw; PFN: Proximal Femoral Nail; PFNA: Proximal Femoral Nail Antirotation; PCCP: Percutaneous Compression Plate; Targon PF: Targon Proximal Femoral; LISS: Less Invasive Stabilization System.

*Statistic significant difference; —: Not appliance.

**Table 5:** Meta-analysis of procedure measure

Intervention	Operative time (min)			Blood loss (mL)			Hospital stay (day)		
	N (n)	MD (95%CI)	I^2^	N (n)	MD (95%CI)	I^2^	N (n)	MD (95%CI)	I^2^
**GN vs.**									
SHS	10 (1817)	−0.49 (−7.40,6.41)	94%	4 (1114)	−21.41 (−97.2,54.37)	79%	7 (909)	0.64 (−1.61,1.15)	0%
ACE nails	2 (185)	−7.16 (−14.99,0.68)	85%	1 (73)	−13.0 (−19.22,−6.78)*	—	—	—	—
PFN	1 (424)	0.0 (−0.38,0.38)	—	1 (424)	67.0 (64.01,69.99)*	—	1 (374)	−2.7 (−2.96,−2.44)*	—
PFNA	2 (197)	4.45 (3.73,5.17)*	0%	1 (136)	55.3 (50.53,60.07)*	—	2 (197)	0.20 (0.13,0.27)	0%
PCCP	—	—	—	—	—	—	1 (118)	−0.1 (−0.52,0.32)	—
**SHS vs.**									
PCCP	4 (360)	7.89 (−8.16,23.95)	92%	2 (169)	133.04 (−16.74,282.83)	93%	2 (211)	−0.32 (−2.43,1.78)	0%
IMHS	3 (342)	−8.83 (−25.06,7.39)	85%	2 (235)	62.42 (26.28,98.56)*	0%	2 (237)	−1.00 (−3.37,1.37)	0%
Medoff	2 (666)	−29.50 (−58.89,−0.12)*	95%	2 (666)	−99.05 (−166,−32.1)*	69%	1 (506)	0.00 (−1.33,1.33)	—
PFN	2 (286)	2.30 (−5.04,9.64)	0%	—	—	—	2 (314)	−0.07 (−1.68,1.54)	0%
PFNA	3 (308)	14.0 (−16.50,44.50)	99%	2 (227)	253.86 (237.47,270.25)*	0%	1 (106)	0.40 (−0.23,1.03)	—
Targon PF	1 (600)	−3.0 (−5.0,−1.0)*	—	—	—	—	1 (600)	−2.5 (−10.61,5.61)	—
Holland nail	1 (190)	−13.7 (−19.25,−8.15)*	—	1 (190)	82.0 (37.81,126.91)*	—	—	—	—
**PFNA vs.**									
LISS	2 (151)	−26.78 (−32.8,−20.75)*	0%	2 (151)	−25.11 (−62.59,12.07)	0%	2 (151)	0.51 (−3.99,5.01)	<0.001
Targon PF	1 (80)	−18.5 (−30.63,−6.37)*	—	—	—	—	—	—	—
PCCP	1 (90)	13.5 (7.54,19.46)*	—	1 (90)	37.5 (20.88,54.12)	—	1 (90)	0.8 (−0.84,2.44)	—

N: the number of studies; n: number of patients.

GN: Gamma Nail; IMHS: Intramedullary Hip Screw; SHS: Sliding Hip Screw; PFN: Proximal Femoral Nail; PFNA: Proximal Femoral Nail Antirotation; PCCP: Percutaneous Compression Plate; Targon PF: Targon Proximal Femoral; LISS: Less Invasive Stabilization System.

*Statistically significant difference.
