# Supplementary material for: Internal fixation treatments for intertrochanteric fracture: a systematic review and meta-analysis of randomized evidence
Source: Sci Rep. 2015 Dec 11;5:18195. doi: 10.1038/srep18195 (PMC4676068; doi:10.1038/srep18195)
Supplement: Supplementary Information [file srep18195-s1.doc]

**Internal fixation treatments** **for intertrochanteric fracture: a** **systematic review and meta-analysis of randomized evidence**

Jiajie Yu^1^, Chao Zhang^2^, Ling Li^1,3^, Joey S.W. Kwong^1,3^, Li Xue^4^, Xiantao Zeng^5^, Li Tang^6^,Youping Li^*1,3^, Xin Sun*^1,2,3^

Affiliations

1. Chinese Evidence-based Medicine Center, West China Hospital, Sichuan University, Chengdu, China, 610041
2. Center for Evidence-based Medicine and Clinical Research, Taihe Hospital, Hubei University of Medicine, Hubei, China, 442000
3. Clinical Research and Evaluation Unit, West China Hospital, Sichuan University,610041
4. Department of orthopedics, The third people’s hospital of Chengdu, Chengdu, China,610031
5. Center for Evidence-based and Translational Medicine, Zhongnan Hospital, Wuhan University, Wuhan, China,430071
6. School of Public Health, Curtin University, Perth, WA, Australia, 6845

Corresponding authors

Youping Li, No. 37 Guo Xue Xiang, Chengdu, Sichuan, 610041, China, +8618980601792, yzmylab@hotmail.com

Xin Sun, No. 37 Guo Xue Xiang, Chengdu, Sichuan, 610041, China, +8618980606047, sunx79@hotmail.com

**Supplementary information**

Appendix 1 Search strategy

Appendix 2 Risk of bias of included studies

Appendix figures Forest plots for functional measures, adverse events and procedure measures

Appendix 1 Search strategy

**PubMed：**

#1 hip fractures [Mesh]

#2 hip fracture [tiab]

#3 extracapsular fracture*[tiab]

#4 intertrochanteric fracture*[tiab]

#5 (hip[tiab] OR hips[tiab] OR trochant*[tiab] OR pertrochant*[tiab] OR intertrochant*[tiab] OR extracapsular*[tiab]) AND fracture [tiab]

#6 #1 OR #2 OR #3 OR #4 OR #5

#7 "Internal Fixators"[Mesh]

#8 "Bone Plates"[Mesh]

#9 "Fracture Fixation, Internal"[Mesh]

#10 "Bone Plates"[Mesh]

#11 "Bone Nails"[Mesh]

#12 "Bone Screws"[Mesh])

#13 internal fixation device*[tiab]

#14 internal fixator*[tiab]

#15 bone plate*[tiab]

#16 internal fracture fixation*[tiab]

#17 fracture osteosynthes*[tiab]

#18 bone nail*[tiab]

#19 bone screw*[tiab]

#20 pin[tiab] OR pins[tiab]

#21 nail[tiab] OR nails[tiab] OR nailing*[tiab]

#22 plate[tiab] OR plates[tiab]

#23 rod[tiab] OR rods[tiab]

#24 screw[tiab] OR screws[tiab]

#25 #7 OR #8 OR #9 OR #10 OR #11 OR #12 OR #13 OR #14 OR #15 OR #16 OR #17 OR #18 OR #19 OR #20 OR #21 OR #22 OR #23 OR #24

#26 Randomized controlled trial[pt] OR Controlled clinical trial[pt] OR Randomized[tiab] OR Placebo[tiab] OR Clinical Trials as Topic[Mesh] OR Randomly[tiab] OR Trials[ti]

#27 #6 AND #25 AND #26

#28 animals [mh] NOT humans [mh]

#29 #27 NOT #28

**EMBASE (via OVID)**

1 exp hip fractures/

2 (hip fracture$ or intertrochanteric fracture$ or extracapsular fracture$).tw.

3 (hip or hips or trochant$ or pertrochant$ or intertrochant$ or extracapsular$).tw.

4 fractur$.tw.

5 3 and 4

6 1 or 2 or 5

7 exp internal fixator/

8 exp bone plate/

9 exp fracture fixation/

10 exp bone nail/

11 exp bone screw/

12 (internal fixation device$ or internal fixator$ or internal fixation system$ or internal fracture fixation$).tw.

13 (bone plate$ or fixation plate$ or bone nail$ or bone screw$ or pin? or nail? or nailing$ or plate? or rod? or screw?).tw.

14 7 or 8 or 9 or 10 or 11 or 12 or 13

15 clinical trial (topic)/

16 controlled clinical trial/

17 Randomized controlled trial (topic)/

18 random.ab.
19 placebo.ab.

20 trial.ab.

21 15 or 16 or 17 or 18 or 19 or 20

22 6 and 14 and 21

23 limit 22 to english

24 limit 23 to humans

**CENTRAL (OVID online)**

1 exp hip fractures/

2 (hip fracture$ or intertrochanteric fracture$ or intertrochanteric fracture$ ).tw.

3 (hip? or femur$ or femoral$ or trochant$ or pertrochant$ or intertrochant$ or subtrochant$ or intracapsular$ or extracapsular$ or acetabul$).tw.

4 fractur$.tw.

5 3 and 4

6 1 or 2 or 5

7 exp internal fixator/

8 exp bone plate/

9 exp fracture fixation/

10 exp bone nail/

11 exp bone screw/

12 (internal fixation device$ or internal fixator$ or internal fixation system$ or internal fracture fixation$).tw.

13 (bone plate$ or fixation plate$ or bone nail$ or bone screw$ or pin? or nail? or nailing$ or plate? or rod? or screw? or fixation$).tw.

14 7 or 8 or 9 or 10 or 11 or 12 or 13

15 6 and 14

Appendix 2 Risk of bias of included studies

| Study | Random sequence generation | Allocation concealment | Blinding of participants | Blinding of clinicians | Blinding of outcome assessor | Free of imcomplete outcome data | Free of selective reporting | Standardize surgical procedure | Surgeons were experienced with operation |
| --- | --- | --- | --- | --- | --- | --- | --- | --- | --- |
| Adams | Unclear | Unclear | High | High | Low | Low | Low | Unclear | Low |
| Ahrengart | Unclear | Unclear | High | High | Unclear | Low | Low | Unclear | Low |
| Aktselis | High | High | Unclear | Unclear | Unclear | Low | Low | Unclear | Unclear |
| Barton | Low | Low | High | High | Unclear | unclear | Low | Unclear | Low |
| Bridle | Unclear | Unclear | Unclear | Unclear | Unclear | Low | Low | Unclear | Low |
| Hoffman | Low | Low | Unclear | Unclear | Low | Unclear | Low | Unclear | Low |
| Kukla | Unclear | Unclear | Unclear | Unclear | Unclear | Low | Low | Unclear | Low |
| Leung | High | Unclear | Unclear | Unclear | Unclear | Low | Low | Unclear | High |
| O'Brien | Unclear | Unclear | Unclear | Unclear | Unclear | High | Low | Low | Unclear |
| Ovesen | Low | Unclear | Unclear | Unclear | Unclear | High | Low | Unclear | Low |
| Park,1998 | High | High | Unclear | Unclear | Unclear | Unclear | Low | Unclear | Low |
| Radford | Unclear | Unclear | Unclear | Unclear | Unclear | Low | Low | Unclear | Low |
| Utrilla | High | High | Unclear | Unclear | Unclear | Low | Low | Unclear | Low |
| Efstathopoulos | Unclear | Unclear | Unclear | Unclear | Unclear | Low | Low | Low | Low |
| Grave | Unclear | Unclear | Unclear | Unclear | Unclear | Low | Low | Low | Low |
| Vidyadhara | Low | Unclear | Unclear | Unclear | Low | Unclear | Low | Low | Low |
| Herrera | Unclear | Unclear | Unclear | Unclear | Unclear | Unclear | Low | Unclear | Unclear |
| Schipper | Low | Low | Unclear | Unclear | Unclear | Low | Low | Low | Low |
| Xu(1) | Low | Low | Unclear | Unclear | Unclear | Low | Low | Unclear | Low |
| Vaquero | Low | Low | Unclear | Unclear | Low | High | Low | Low | Unclear |
| Varela-Egocheaga | Unclear | Unclear | Unclear | Unclear | Unclear | Low | Low | Unclear | Low |
| Janzing | Unclear | Unclear | Unclear | Unclear | Unclear | Low | Low | Unclear | Unclear |
| Kosygan | Unclear | Unclear | Unclear | Unclear | Unclear | Low | Low | Unclear | Low |
| Peyser | Low | Low | Unclear | Unclear | Low | High | Low | Unclear | Low |
| Yang | Low | Low | Unclear | Unclear | Unclear | High | Low | Unclear | Low |
| Baumgaertner | Low | Low | Unclear | Unclear | Unclear | Low | Low | Unclear | Unclear |
| Hardy | High | High | Unclear | Unclear | Unclear | Low | Low | Unclear | High |
| Harrington | Unclear | Unclear | Unclear | Unclear | Low | Unclear | Low | Unclear | Low |
| McCormack | Unclear | Unclear | Unclear | Unclear | Unclear | Low | Low | Unclear | Unclear |
| Watson | High | High | Unclear | Unclear | Unclear | Low | Low | Unclear | Low |
| Lunsjo | High | High | Unclear | Unclear | Low | Low | Low | Unclear | High |
| Saudan | Unclear | Unclear | Unclear | Unclear | Unclear | Low | Low | Unclear | Low |
| Garg | Low | Unclear | Unclear | Unclear | Unclear | Unclear | Low | Unclear | Low |
| Xu(2) | Low | Low | Unclear | Unclear | Unclear | Low | Low | Unclear | Low |
| Zou | Unclear | Unclear | Unclear | Unclear | Unclear | Unclear | Low | Unclear | Unclear |
| Little | Low | Unclear | Unclear | Unclear | Low | Low | Low | Unclear | Low |
| Parker | Unclear | Unclear | Unclear | Unclear | Low | Low | Low | Unclear | Low |
| Park,2010 | High | High | Unclear | Unclear | Unclear | Unclear | Low | Unclear | Unclear |
| Guo | Unclear | Unclear | Unclear | Unclear | Unclear | Low | Low | Low | Low |
| Tao | Unclear | Unclear | Unclear | Unclear | Unclear | Low | Low | Unclear | Low |
| Zhou | Low | Unclear | Unclear | Unclear | Unclear | Low | Low | Unclear | Unclear |
| Wild | High | High | Unclear | Unclear | Unclear | Low | Low | Unclear | Low |
| Papasimos | Unclear | Unclear | Unclear | Unclear | Low | Unclear | Low | Unclear | Low |

Appendix figures:


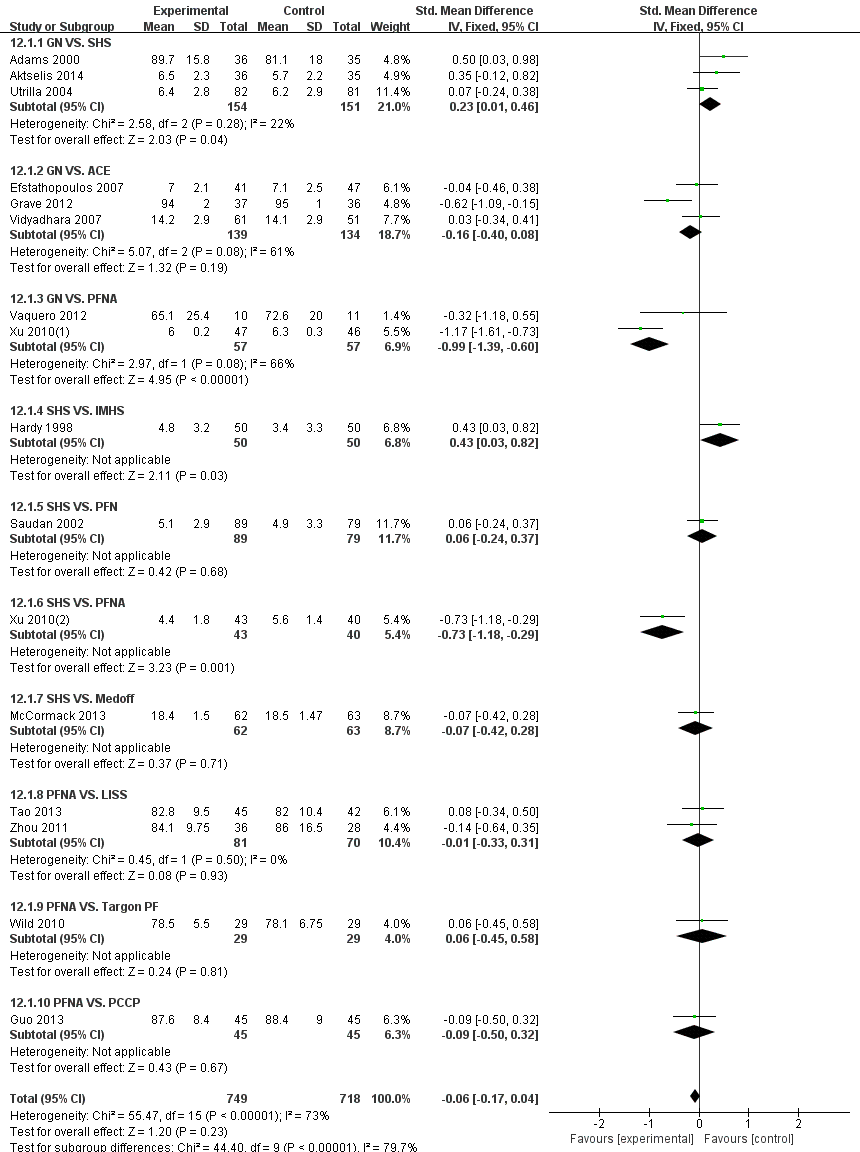


Fig 1 Forest plots for functional scores among different comparisons


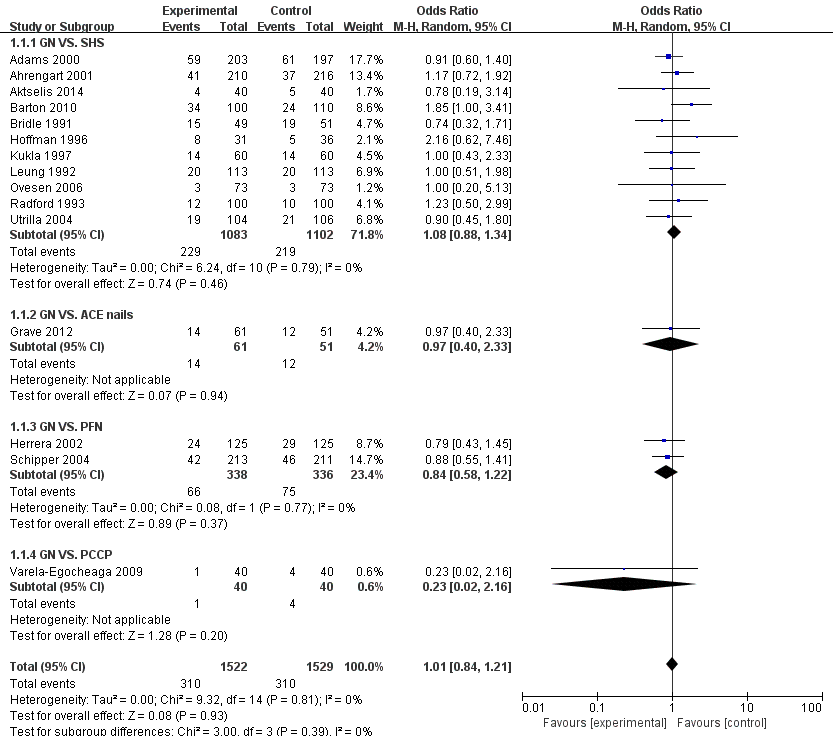


Fig 2 Comparisons between GN and other internal fixation treatments on mortality


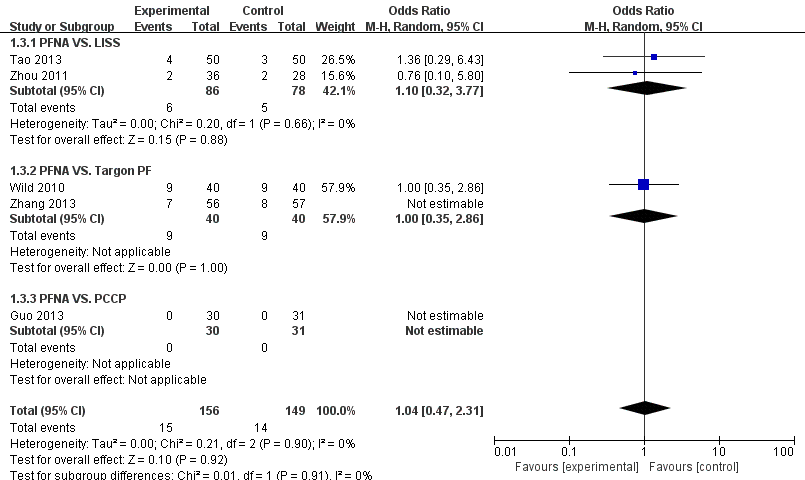


Figure 3 Comparisons between PFNA and other internal fixation treatments on mortality


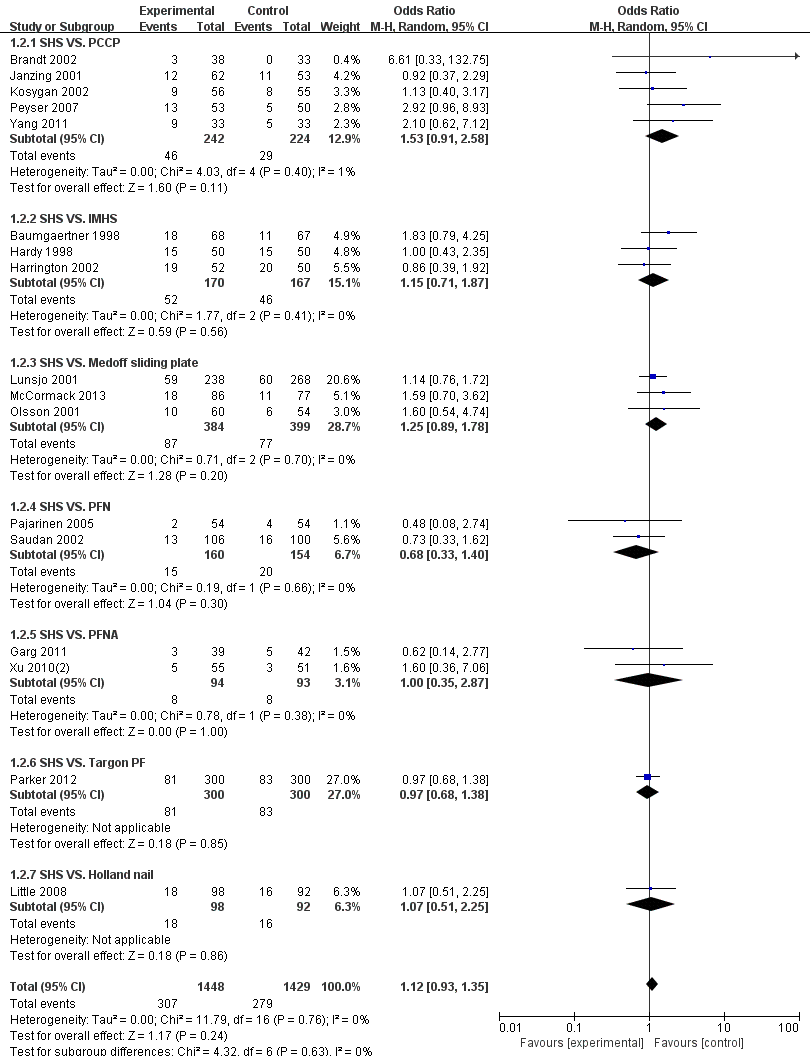


Figure 4 Comparisons between SHS and other internal fixation treatments on mortality


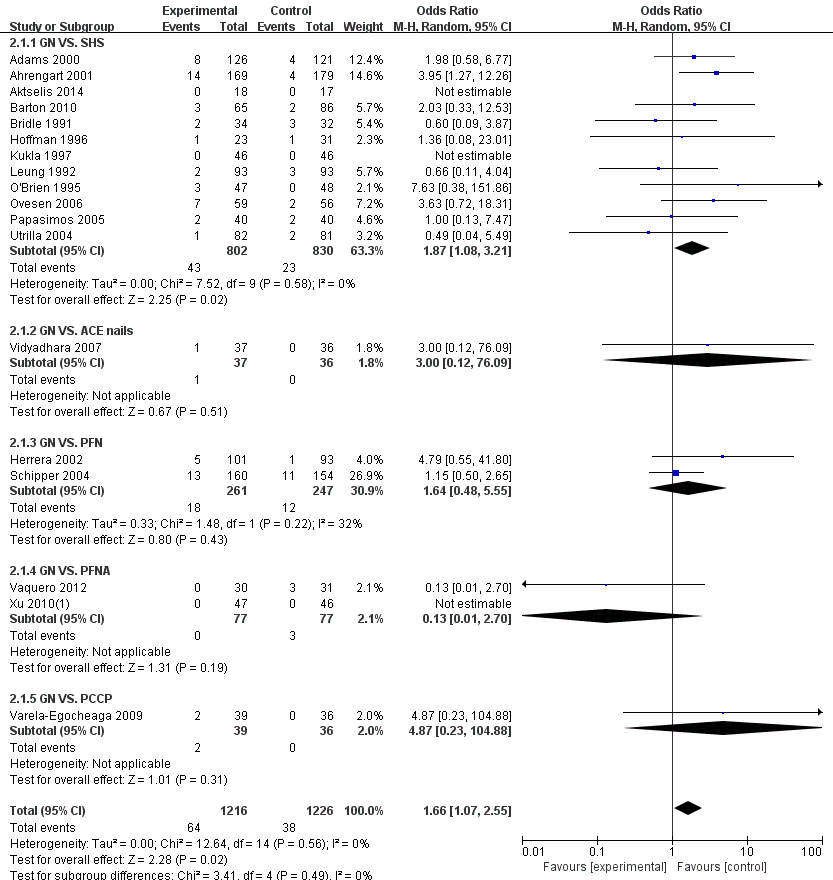


Figure 5 Comparisons between GN and other internal fixation treatments on cut out


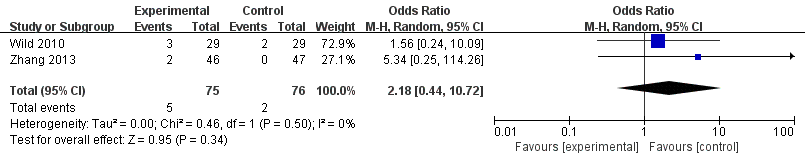


Figure 6 Comparisons between PFNA and other internal fixation treatments on cut out


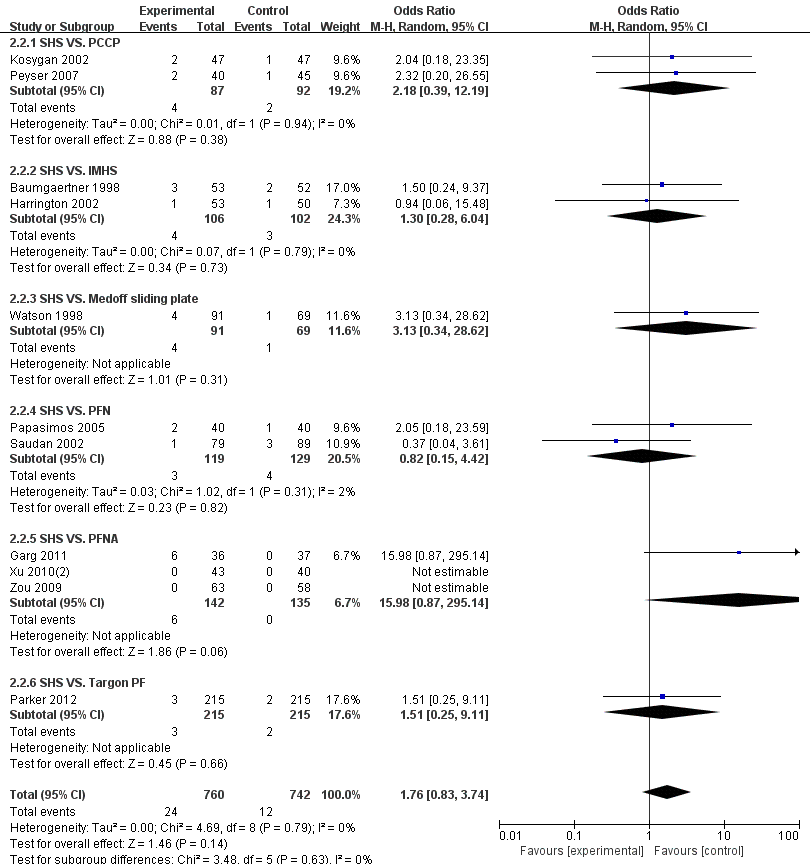


Figure 7 Comparisons between SHS and other internal fixation treatments on cut out


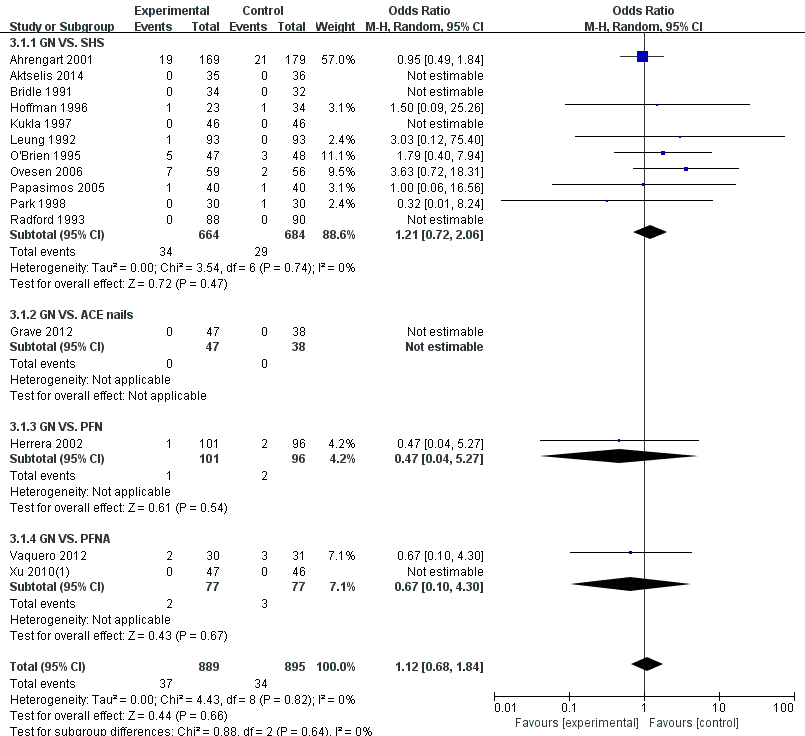


Figure 8 Comparisons between GN and other internal fixation treatments on non-union


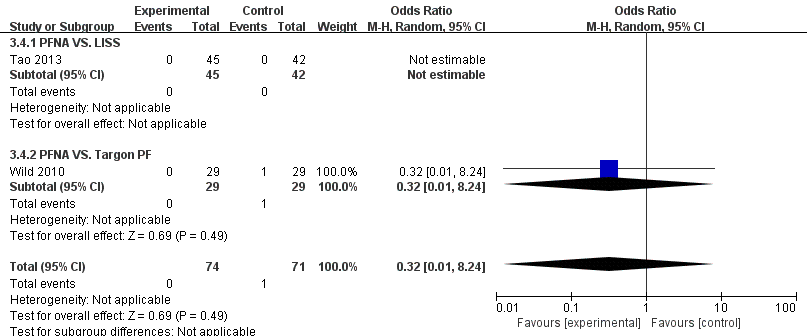


Figure 9 Comparisons between PFNA and other internal fixation treatments on non-union


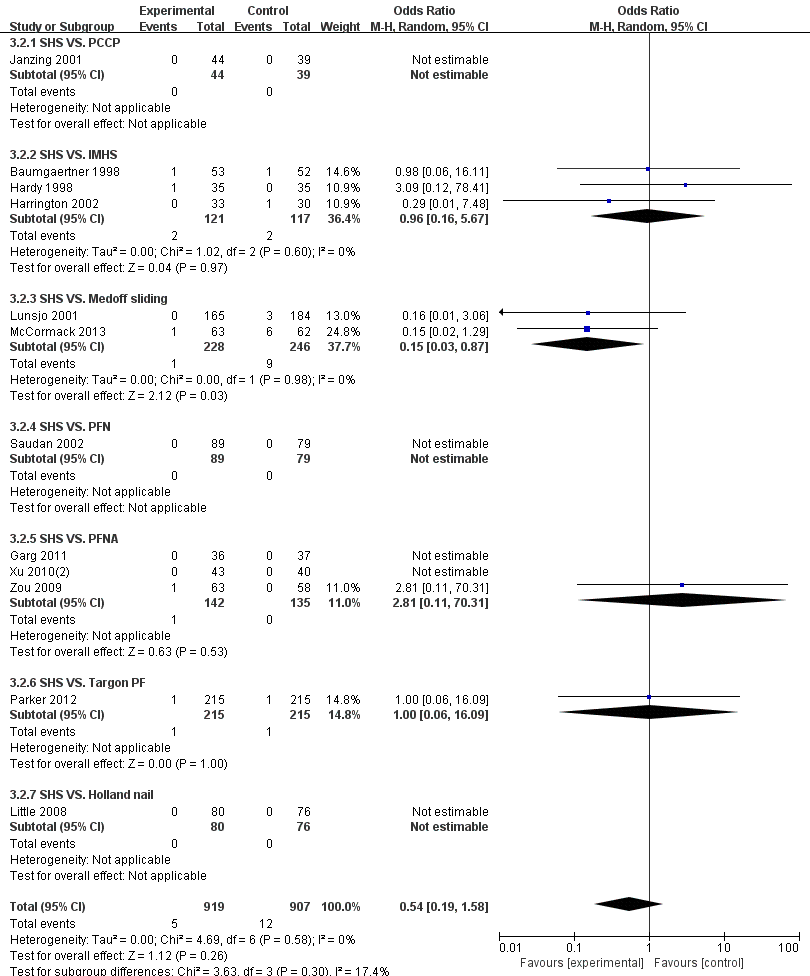


Figure 10 Comparisons between SHS and other internal fixation treatments on non-union


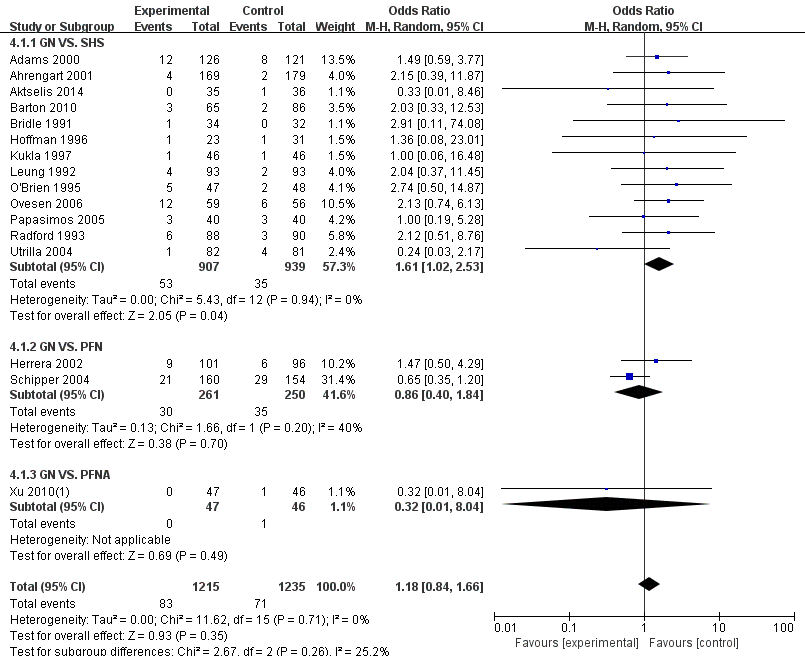


Figure11 Comparisons between GN and other internal fixation treatments on re-operation


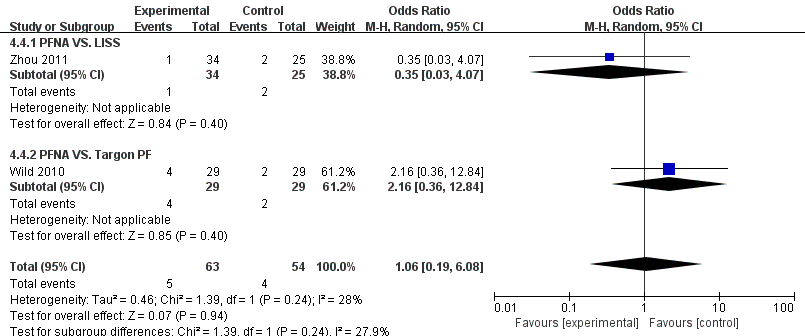


Figure 12 Comparisons between PFNA and other internal fixation treatments on re-operation


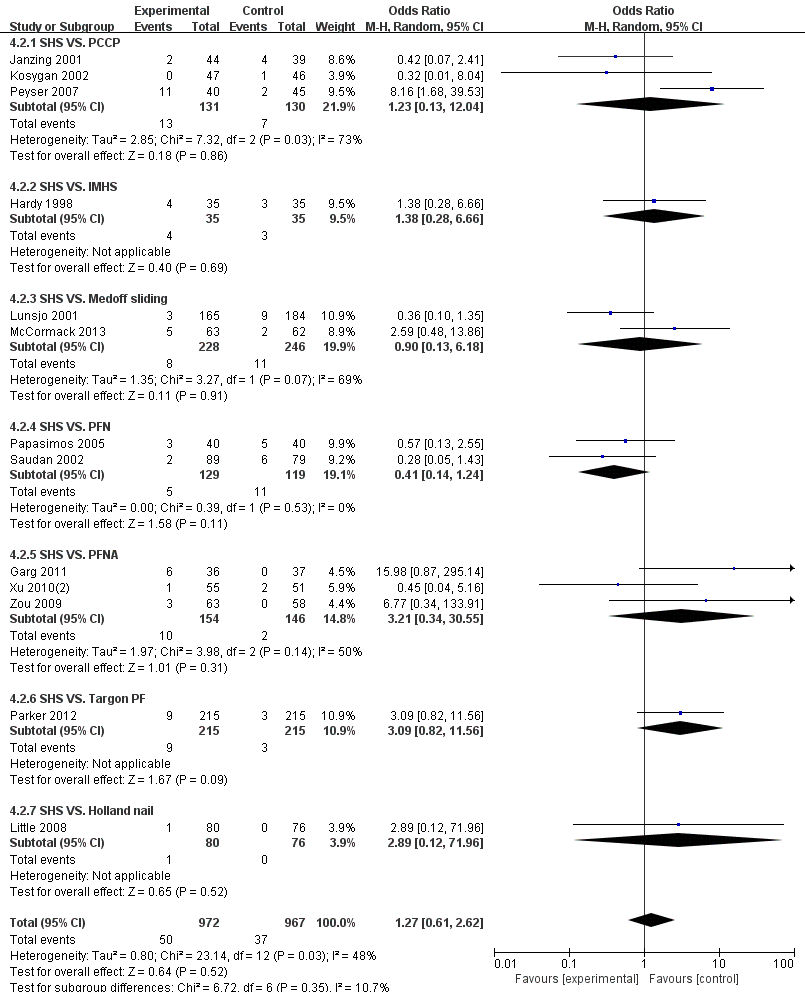


Figure 13 Comparisons between SHS and other internal fixation treatments on re-operation


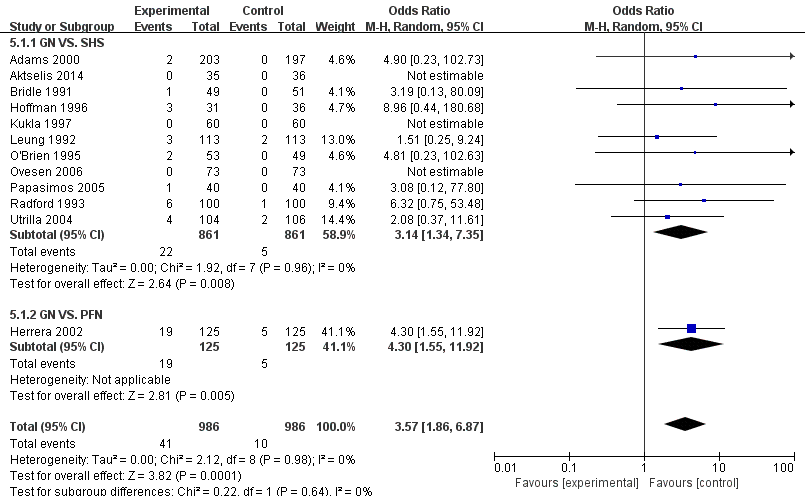


Figure 14 Comparisons between GN and other internal fixation treatments on intra-operation


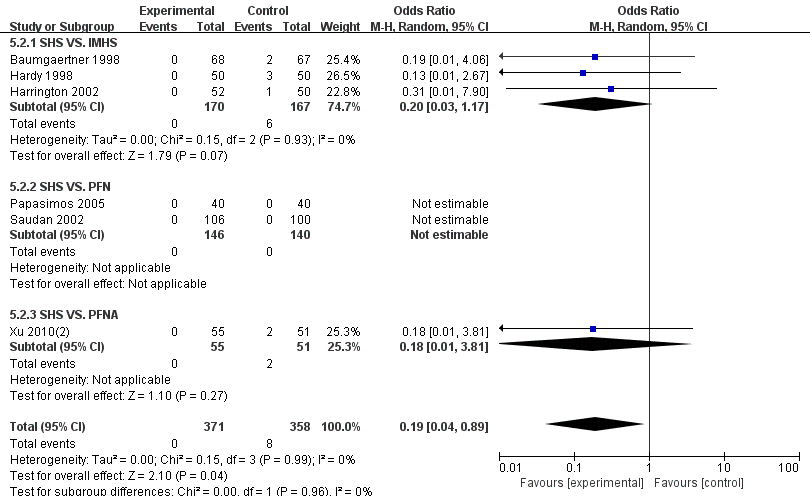


Figure 15 Comparisons between SHS and other internal fixation treatments on intra-operative fracture


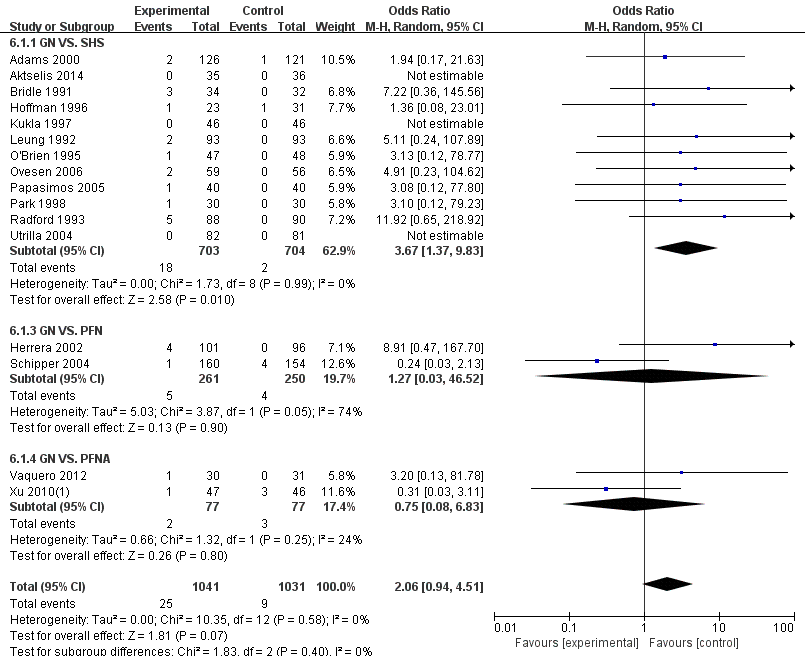


Figure 16 Comparisons between GN and other internal fixation treatments on later fracture


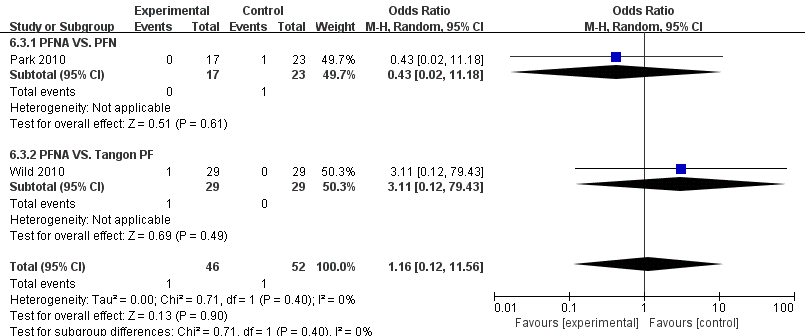


Figure 17 Comparisons between PFNA and other internal fixation treatments on later fracture


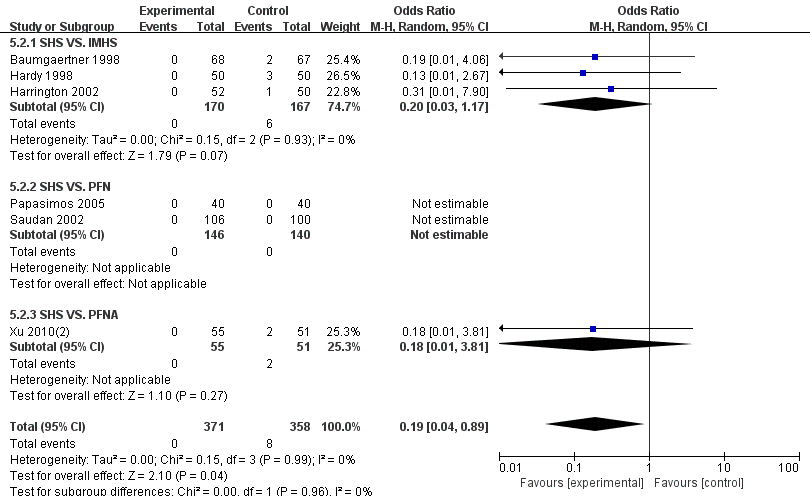


Figure 18 Comparisons between SHS and other internal fixation treatments on later fracture


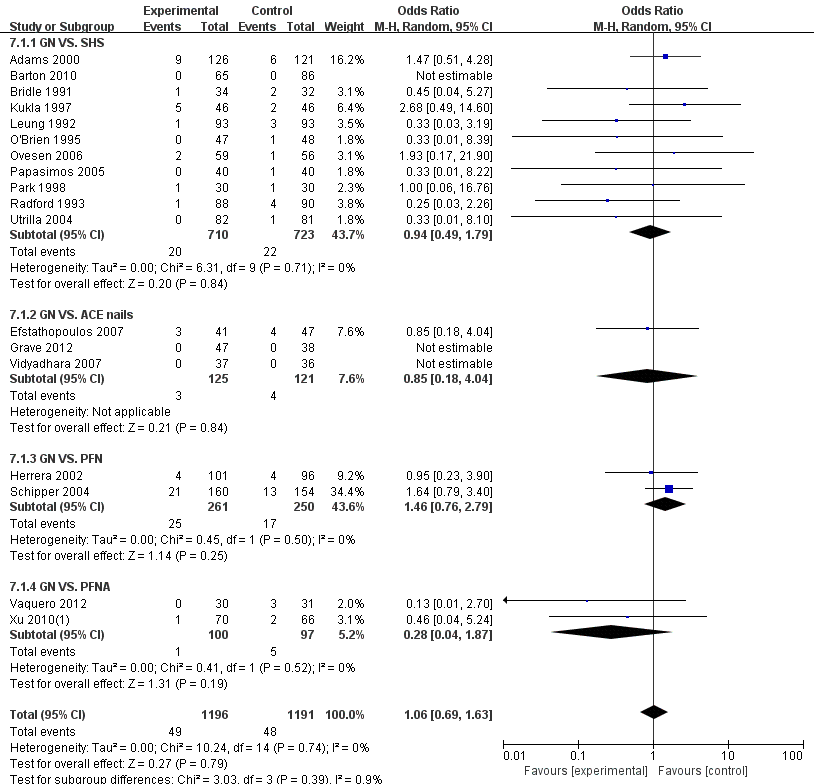


Figure 19 Comparisons between GN and other internal fixation treatments on wound infection


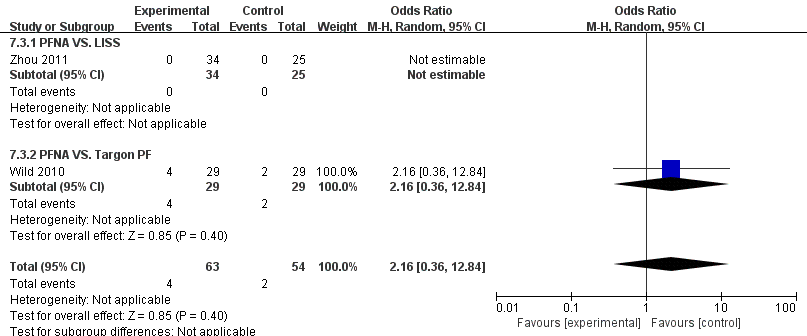


Figure 20 Comparisons between PFNA and other internal fixation treatments on wound infection


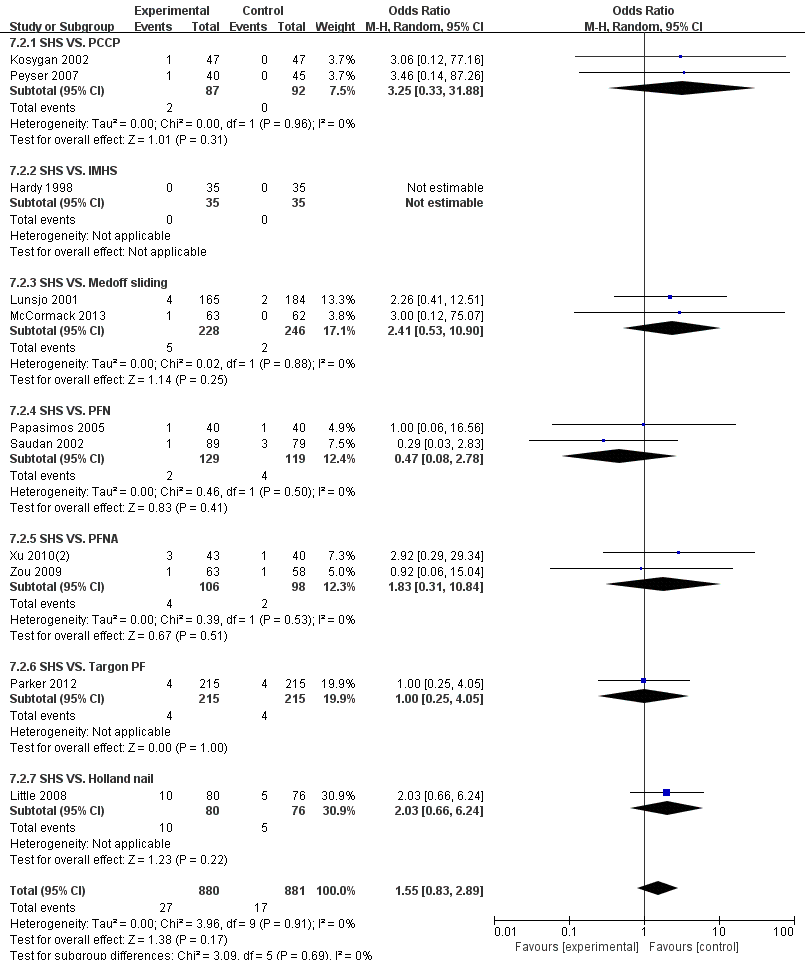


Figure 21 Comparisons between SHS and other internal fixation treatments on wound infection


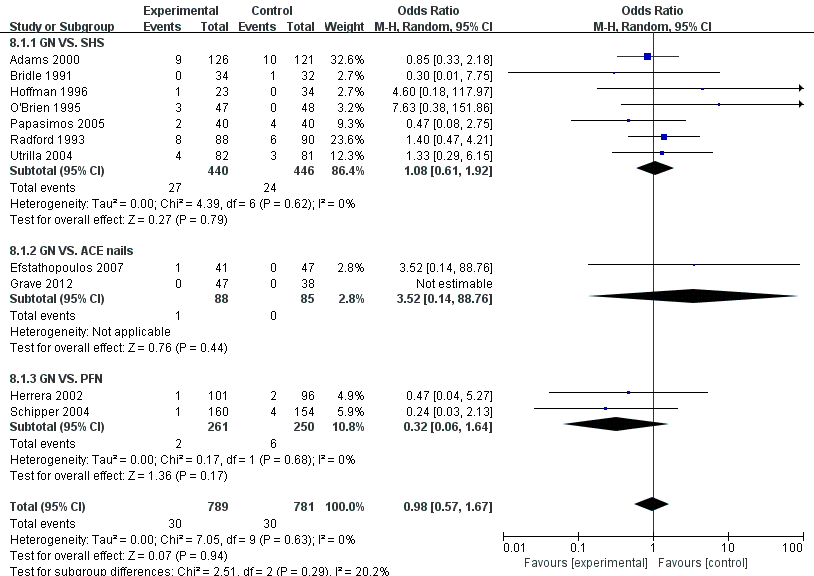


Figure 22 Comparisons between GN and other internal fixation treatments on embolism


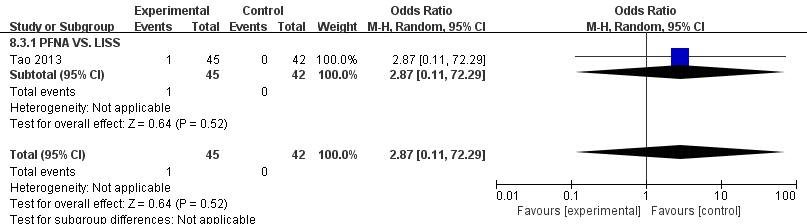


Figure 23 Comparisons between PFNA and other internal fixation treatments on embolism


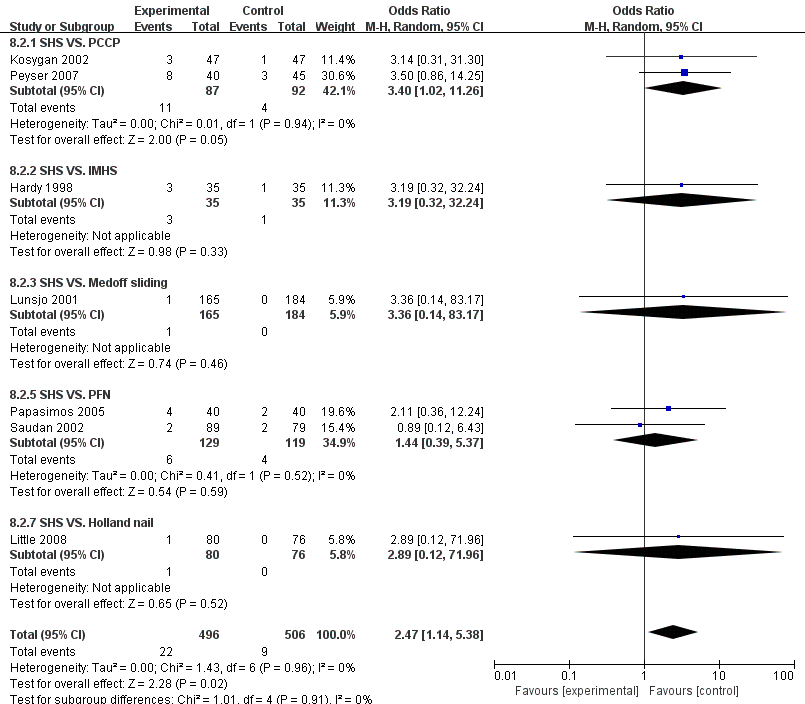


Figure 24 Comparisons between SHS and other internal fixation treatments on embolism


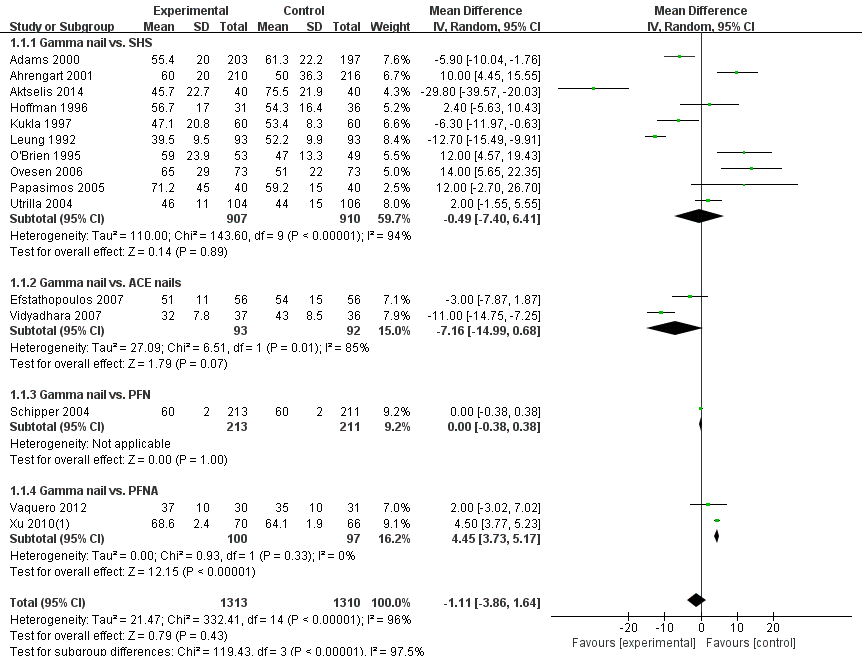


Figure 25 Comparisons between GN and other internal fixation treatments on operative time


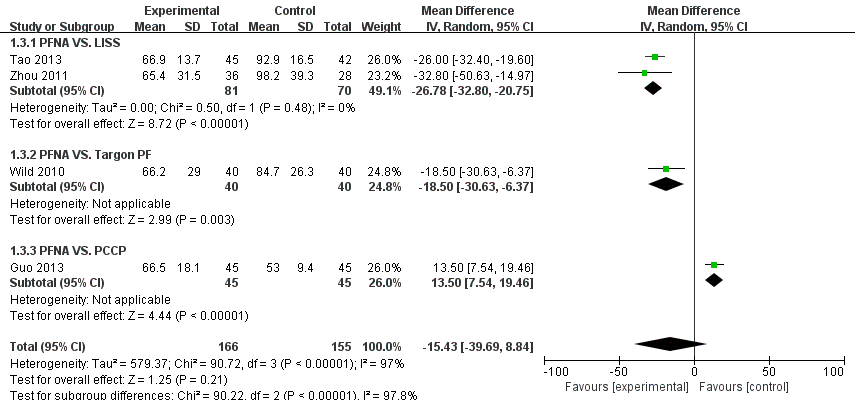


Figure 26 Comparisons between PFNA and other internal fixation treatments on operative time


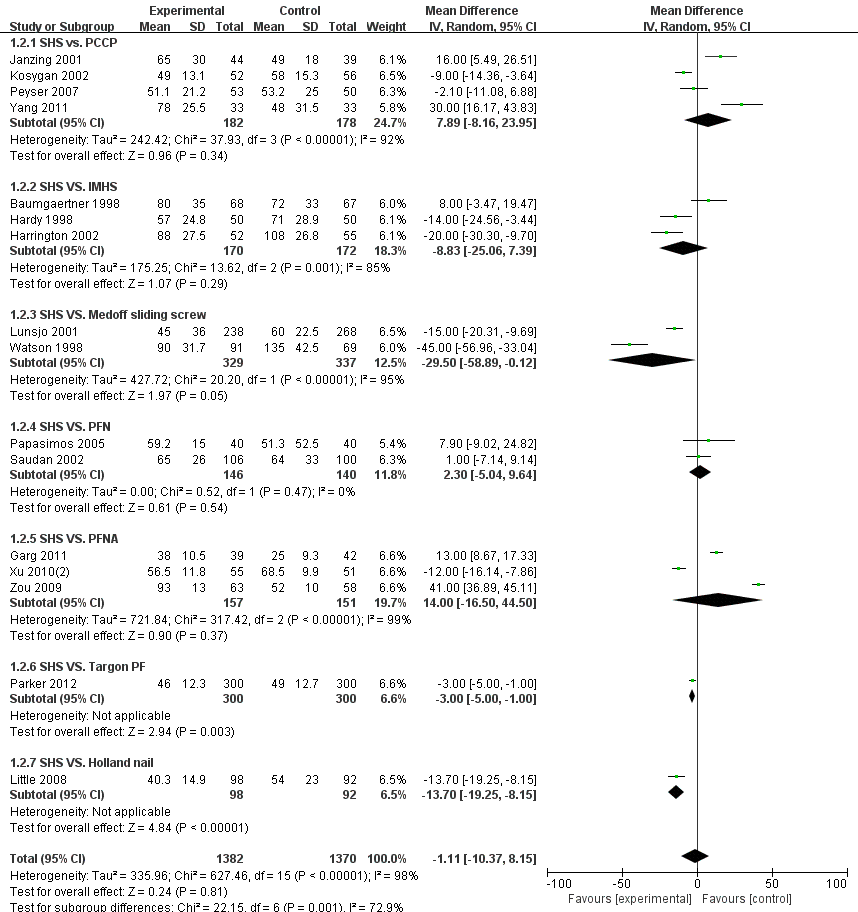


Figure 27 Comparisons between SHS and other internal fixation treatments on operative time


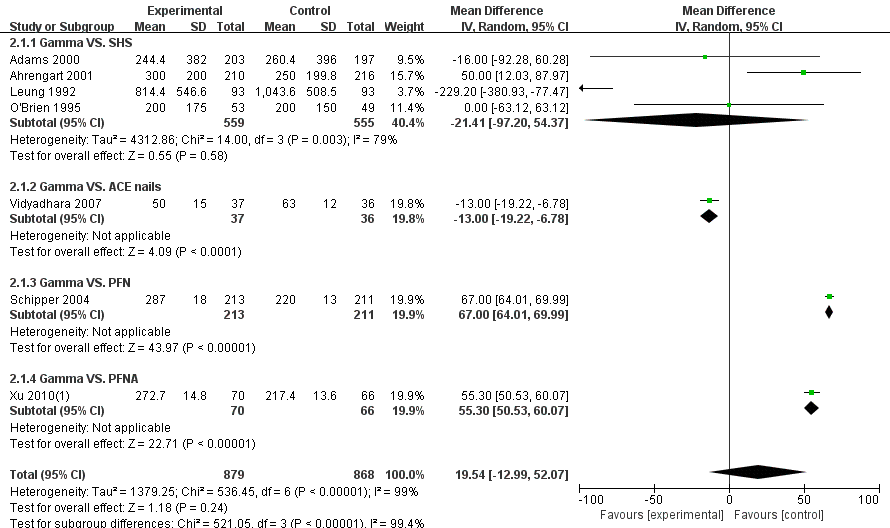


Figure 28 Comparisons between GN and other internal fixation treatments on blood loss


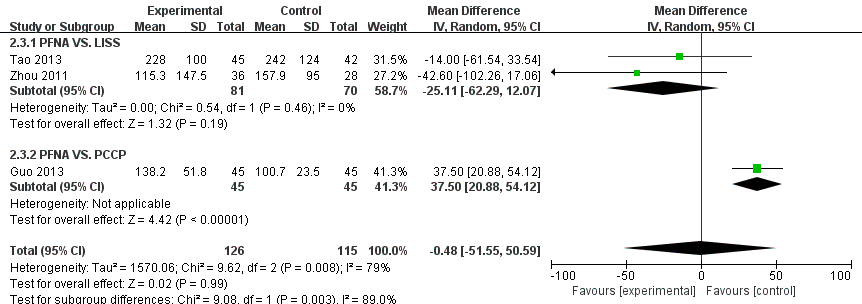


Figure 29 Comparisons between PFNA and other internal fixation treatments on blood loss


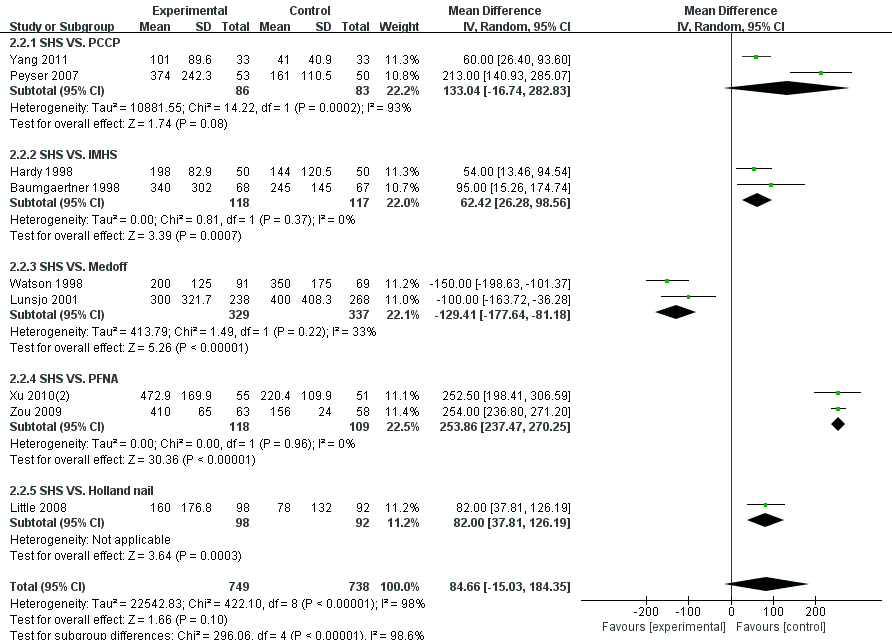


Figure 30 Comparisons between SHS and other internal fixation treatments on blood loss


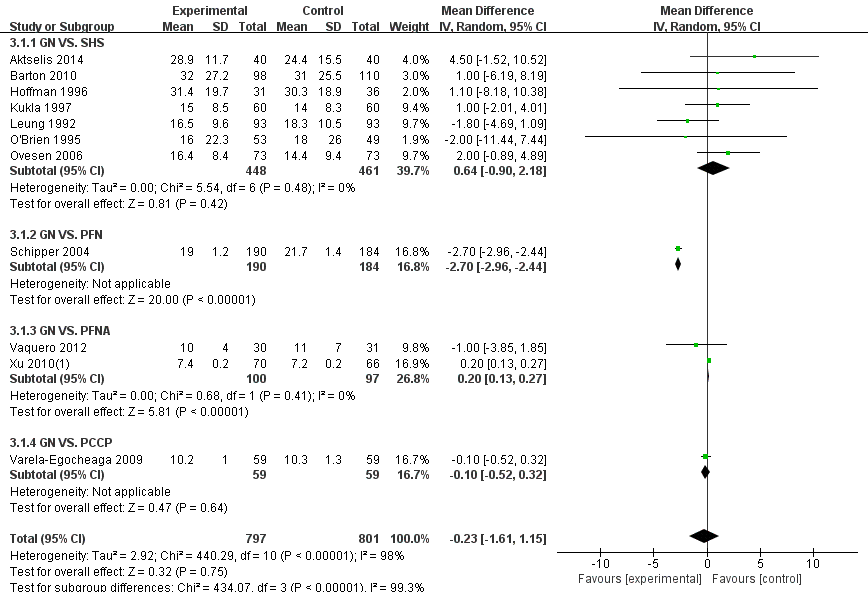


Figure 31 Comparisons between GN and other internal fixation treatments on hospital stay


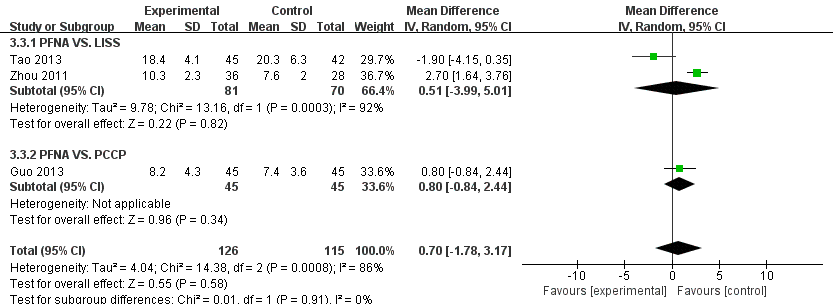


Figure32 Comparisons between PFNA and other internal fixation treatments on hospital stay


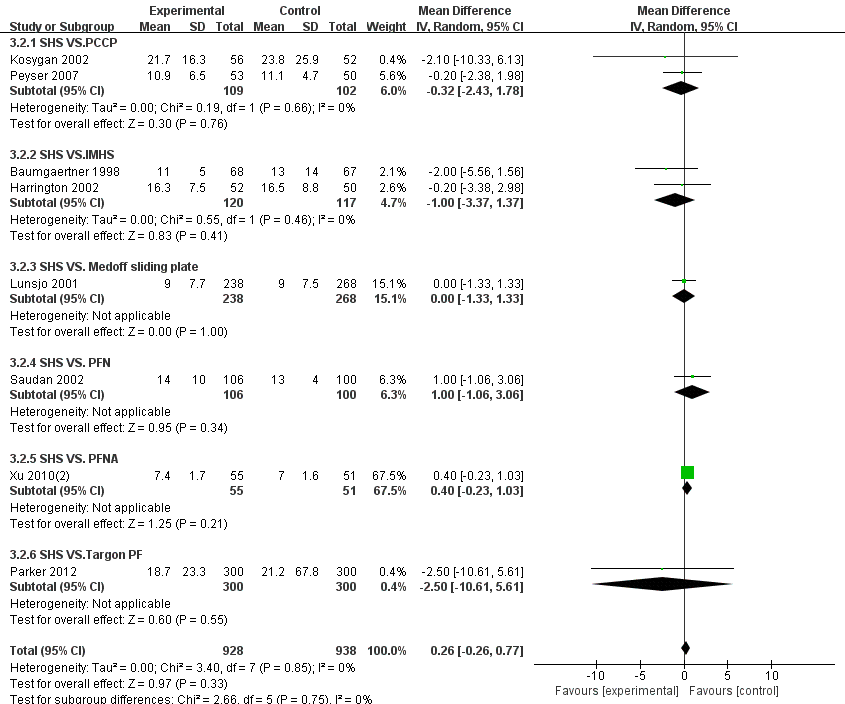


Figure 33 Comparisons between SHS and other internal fixation treatments on hospital stay
